# Supplementary figures and images for: Gene by Environment Interactions reveal new regulatory aspects of signaling network plasticity
Source: PLoS Genet. 2022 Jan 4;18(1):e1009988. doi: 10.1371/journal.pgen.1009988 (PMC8759647; doi:10.1371/journal.pgen.1009988)

**A**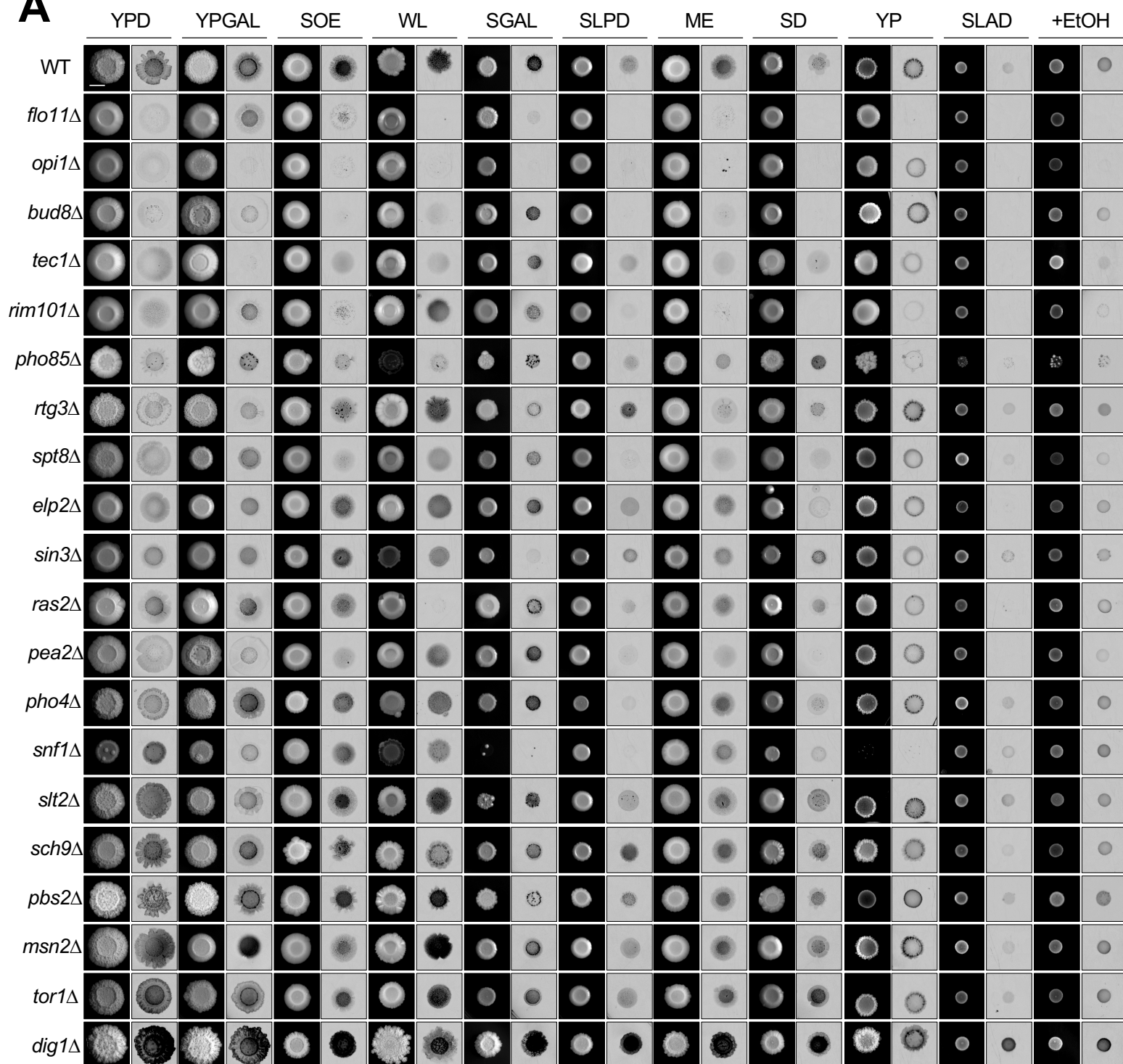**B**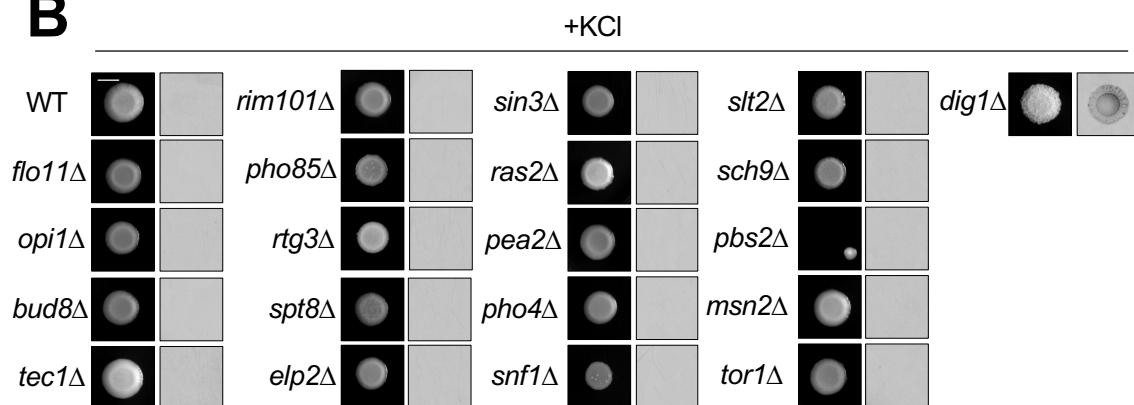**C**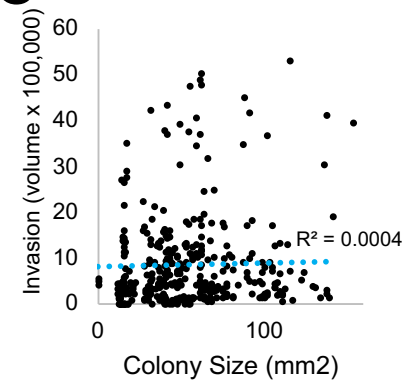

Supplement: S1 Fig — A) PWA on indicated media. First column, cells before wash, second column, inverted images of scars after wash, bar, 0.5 cm. Quantification in S2 Fig. B) PWA on +KCl medium; details in panel A. Only the dig1Δ mutant invades in this environment. C) All replicates on all media for wild type and the mutant strains were plotted for invasion versus colony size (mm2). No correlation between invasion and colony size occurred (R2 = 0.0004). (PDF) [file pgen.1009988.s001.pdf]

**A** ■ YPD ■ YPGAL ■ SOE ■ WL ■ SGAL ■ SLPD ■ ME ■ SD ■ YP ■ SLAD ■ SLAD+EtOH  
NG = No Growth

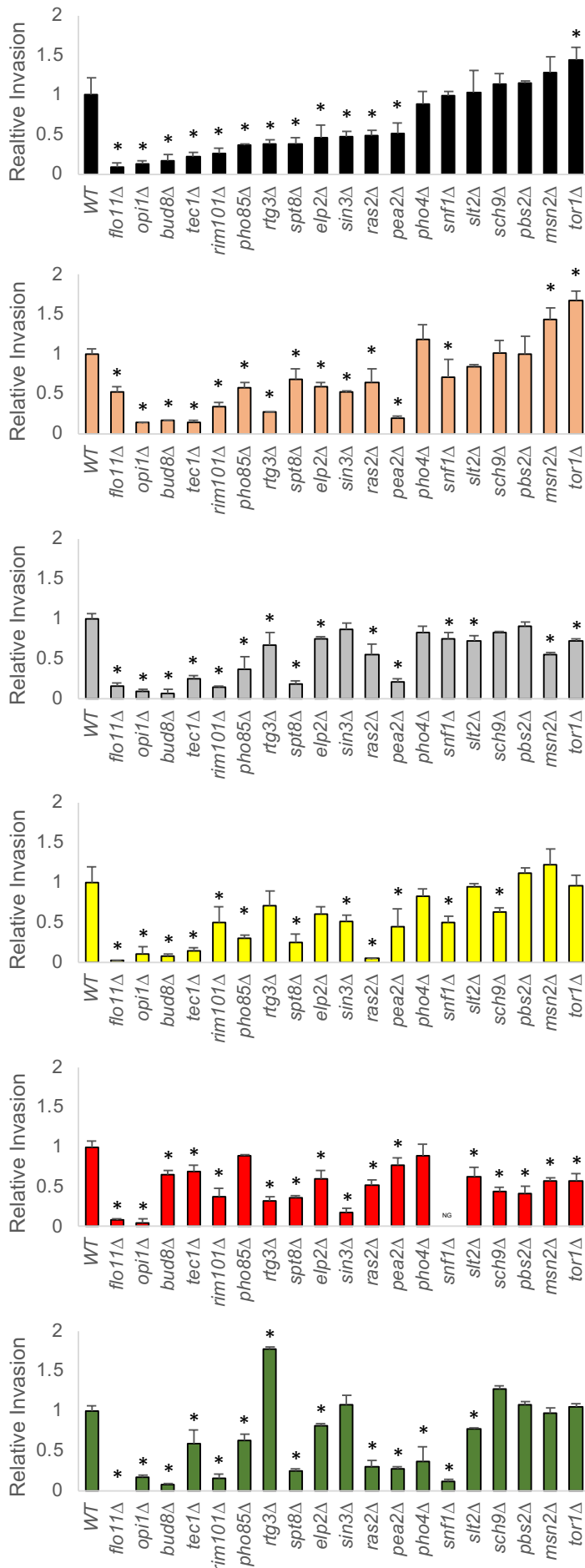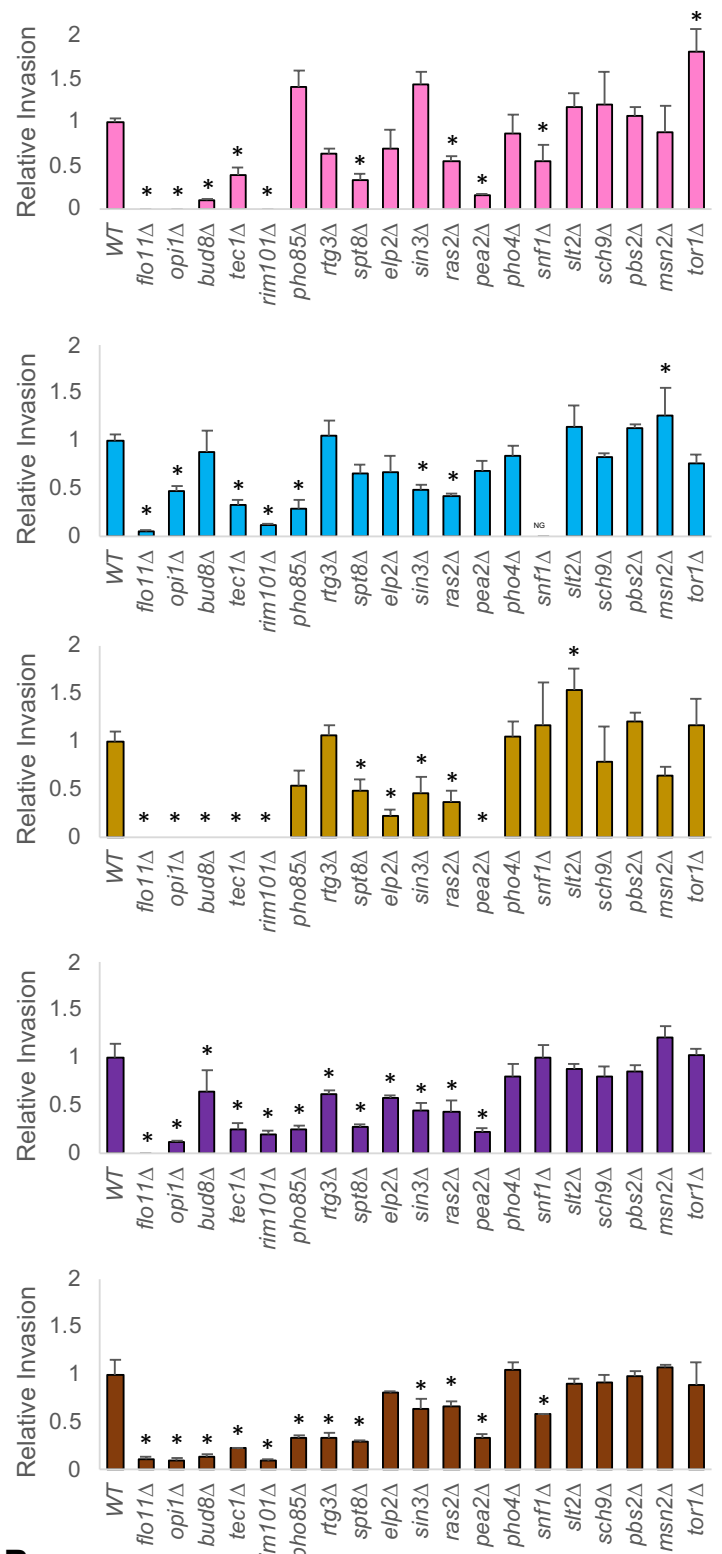

**B**

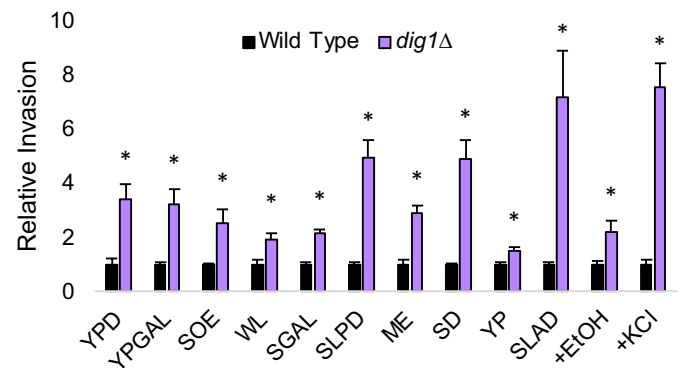

Supplement: S2 Fig — A) PWA; Levels of relative invasion to wild type in indicated media, with wild-type values set to 1. Asterisk, p-value ≤ 0.05, compared to wild type. (Images in S1A Fig) B) Levels of relative invasion for the dig1Δ mutant, with wild-type values set to 1; Asterisk, p-value ≤ 0.05, compared to wild type by Student’s t-test. (PDF) [file pgen.1009988.s002.pdf]

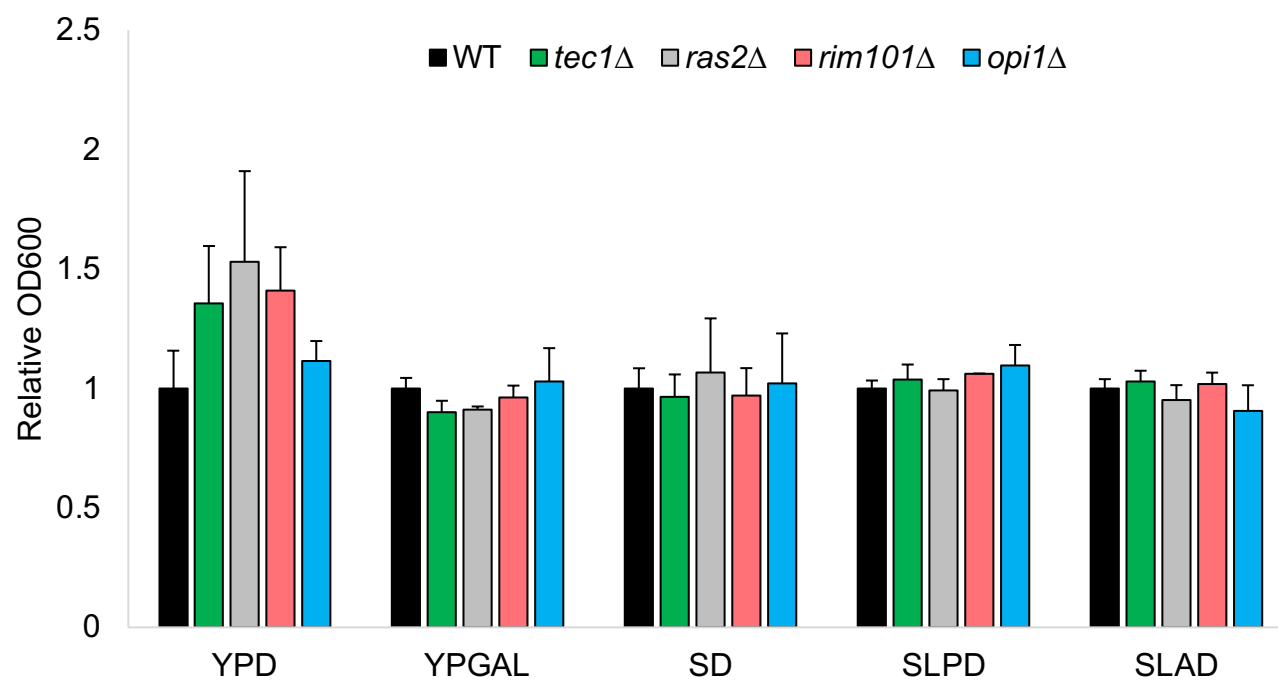

Supplement: S4 Fig — Wild-type cells and the indicated mutants were grown in the indicated media in liquid culture for 16h at 30° by shaking. Cells were washed once in water, and growth was measured by OD at 600nm. For each condition, wild-type values were set to a value of 1. The average of three replicates is reported. Error is reported as standard deviation. (PDF) [file pgen.1009988.s004.pdf]

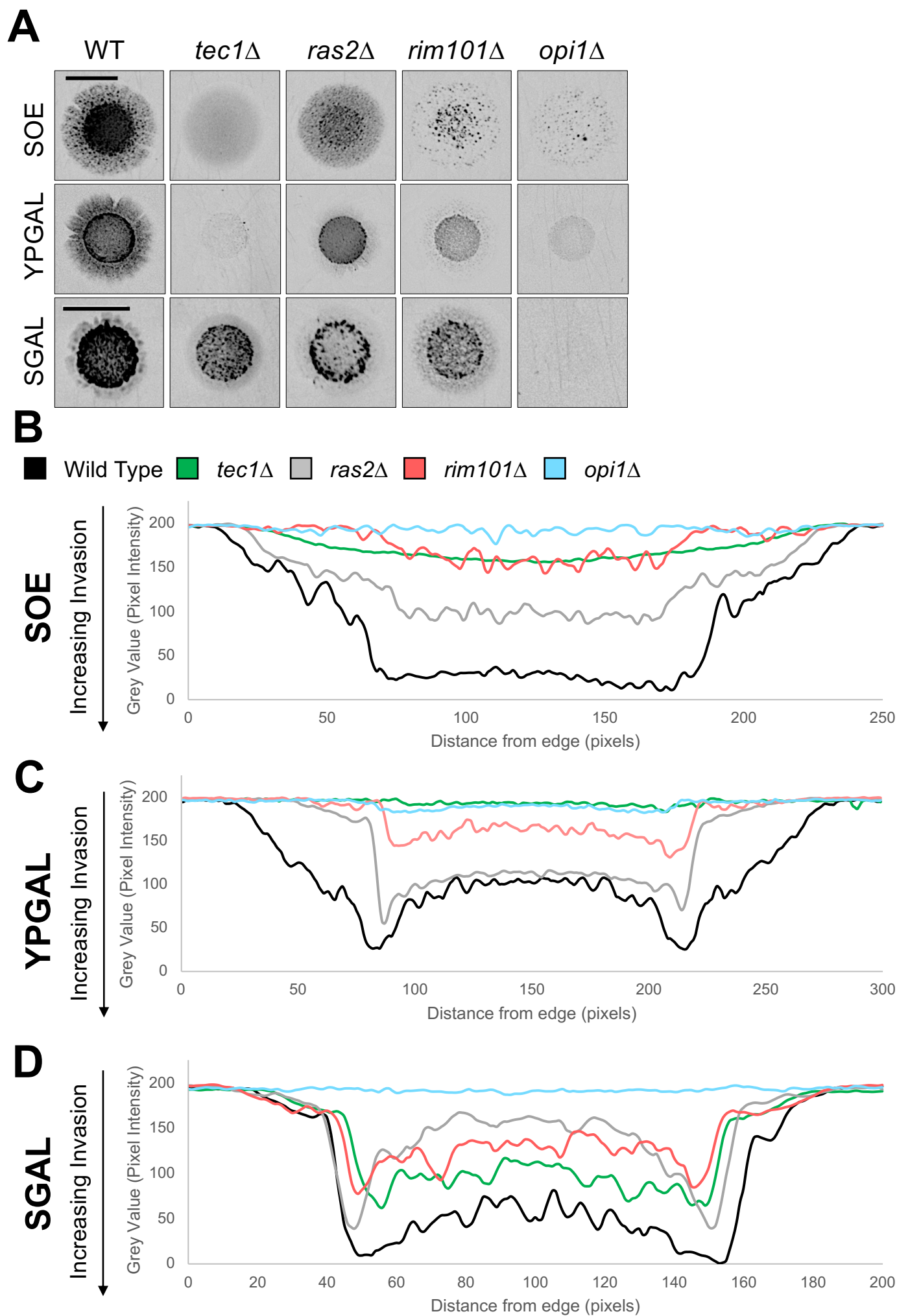

Supplement: S5 Fig — A) PWA. Inverted images of invasive scars shown. Bars, 0.5 cm. B-D) Plot profile of invasive growth across invasive scars. Strains and media are as indicated; examples are shown in panel A. X-axis, distance (in pixels); Y-axis, pixel intensity. (PDF) [file pgen.1009988.s005.pdf]

**A**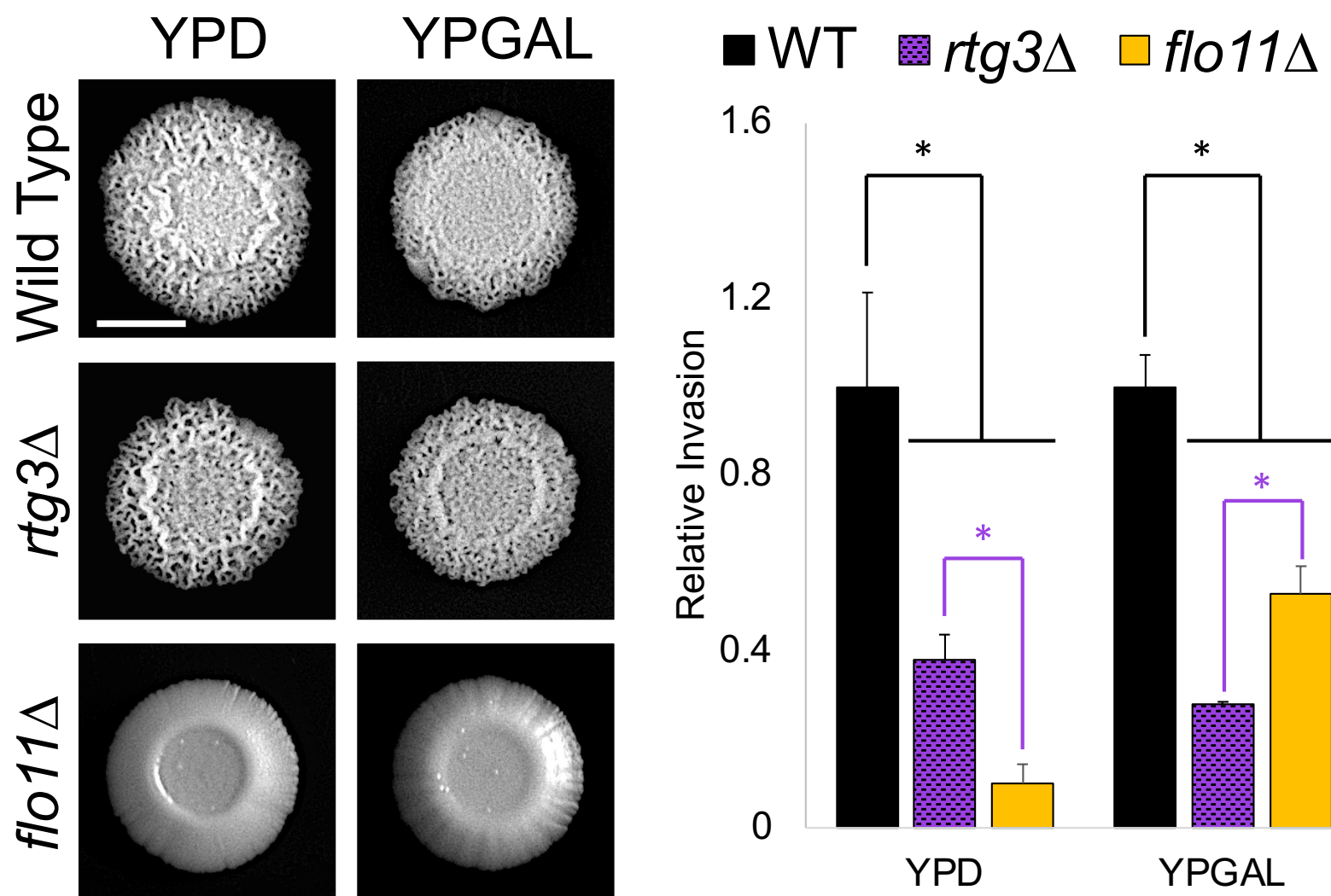**B**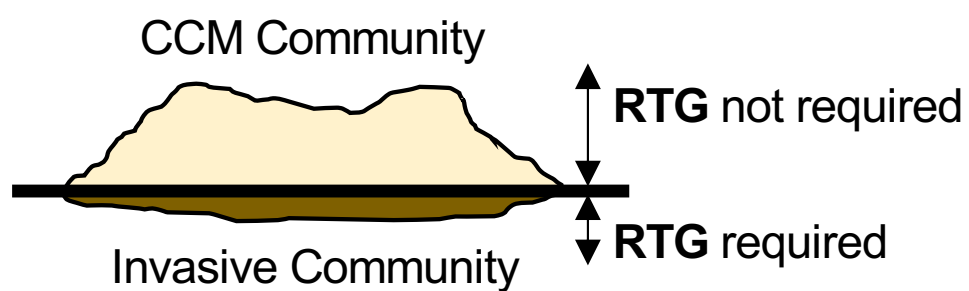

Supplement: S6 Fig — A) Complex-colony morphology analysis. Images of complex-colony morphology are shown. Images are also shown in S1 Fig. Bar graphs, levels of relative invasion, with wild type values set to 1. Black asterisk, p-value < 0.05, compared to wild type. Purple asterisk, p-value < 0.05, comparing the mutants to each other by Student’s t-test. Invasive scar images can be found in S1 Fig. Relative invasion values are also shown in S2 Fig. The rtg3Δ mutant showed decreased invasion on YPD and YPGAL media compared to wild type yet had a similar complex-colony morphology pattern. The flo11Δ mutant showed increased invasion from YPD to YPGAL media with no change in its complex-colony morphology pattern. Moreover, the flo11Δ mutant showed higher invasion but lower complex-colony morphology than the rtg3Δ mutant on YPGAL. B) Model of above and below surface communities depicting the role of RTG. CCM, complex-colony morphology. (PDF) [file pgen.1009988.s006.pdf]

**A**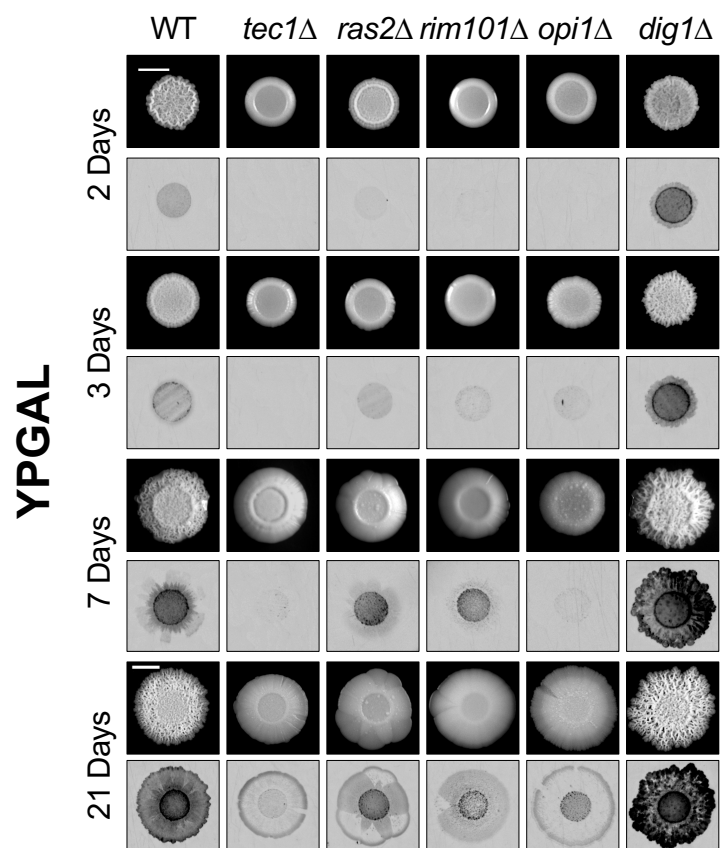**B**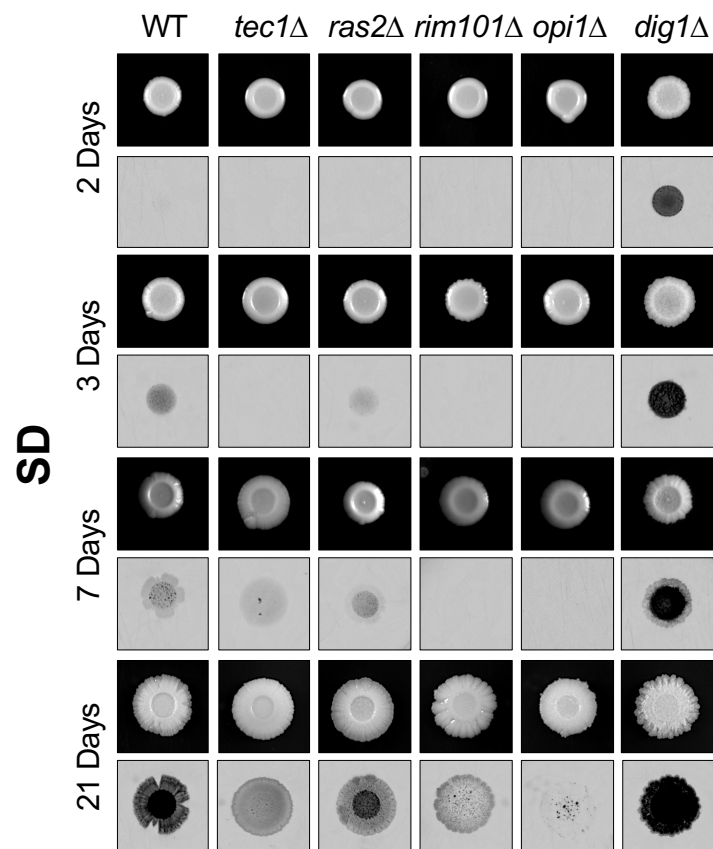**C**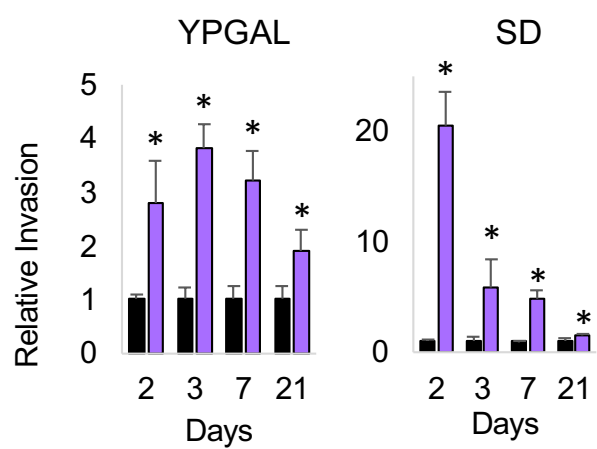**D**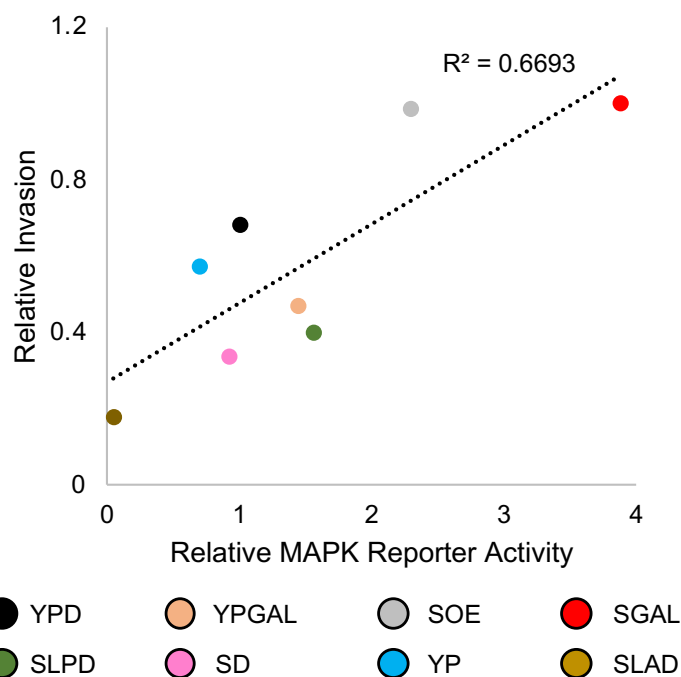**E**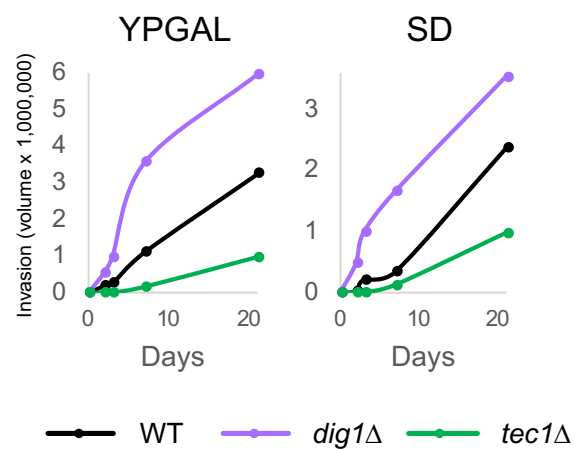

Supplement: S7 Fig — A) PWA. Strains were spotted on YPGAL for the indicated number of days (2 d, 3 d, 7 d, and 21 d). Top row, cells before wash, bottom row, inverted images of scars after wash, bar, 0.5 cm. B) Same as panel A, except on SD medium. C) We found a strong temporal role on SD medium but not YPGAL medium for the dig1Δ mutant. Levels of relative invasion between wild type and the dig1Δ mutant on indicated medium, with wild type values set to 1. Left, YPGAL, right SD. Asterisk, P-value ≤ 0.05, compared to wild type. D) Levels of relative invasive growth versus relative MAPK pathway activity in wild-type cells. E) Levels of invasive growth on YPGAL or SD media over 21 d for wild type (black) and the dig1Δ (purple) and tec1Δ (green) mutants. (PDF) [file pgen.1009988.s007.pdf]

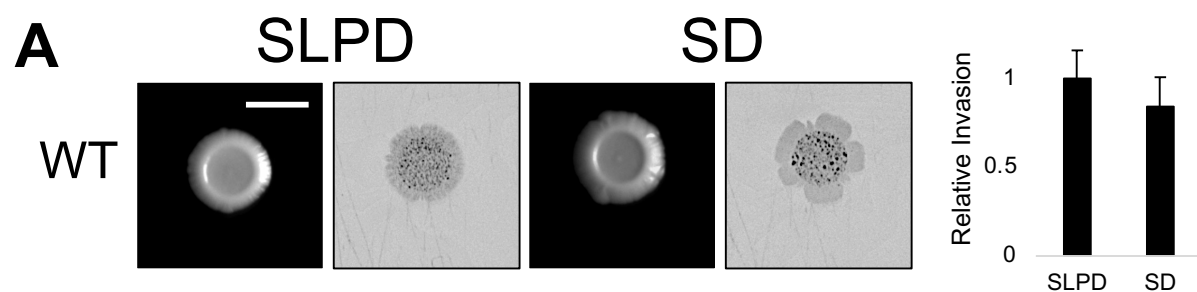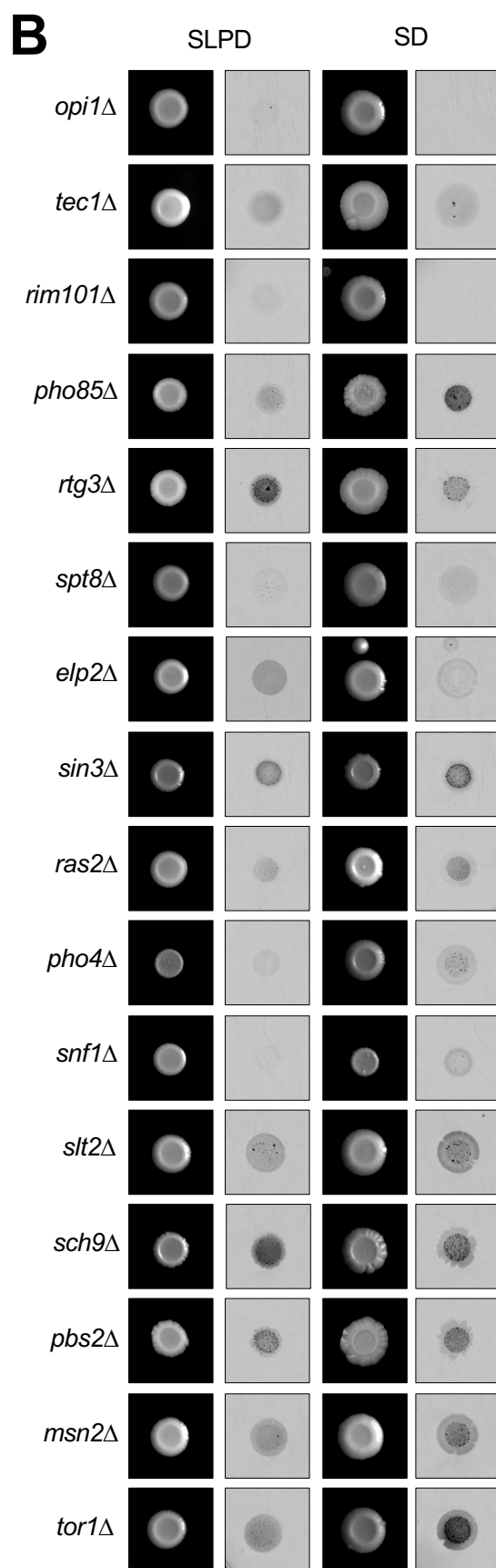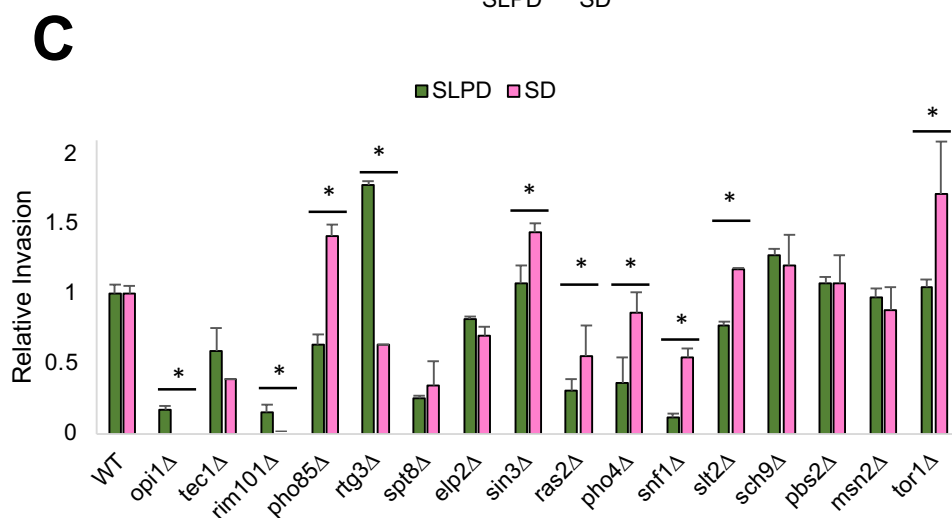

Supplement: S9 Fig — A) PWA of wild type on SLPD and SD media, bar, 0.5 cm. Images are repeats from S1A Fig. Bar graph, levels of relative invasion to SLPD, with SLPD values set to 1. Quantification values are repeated from Fig 1B, except in relative terms. B) PWA on SLPD and SD media. Images are repeats from S1A Fig. C) Levels of relative invasion to wild type, with wild-type values set to 1. Quantification values are from S2 Fig. Asterisk, p-value ≤ 0.05, comparing one strain to itself between SLPD and SD media by Student’s t-test. (PDF) [file pgen.1009988.s009.pdf]

**A**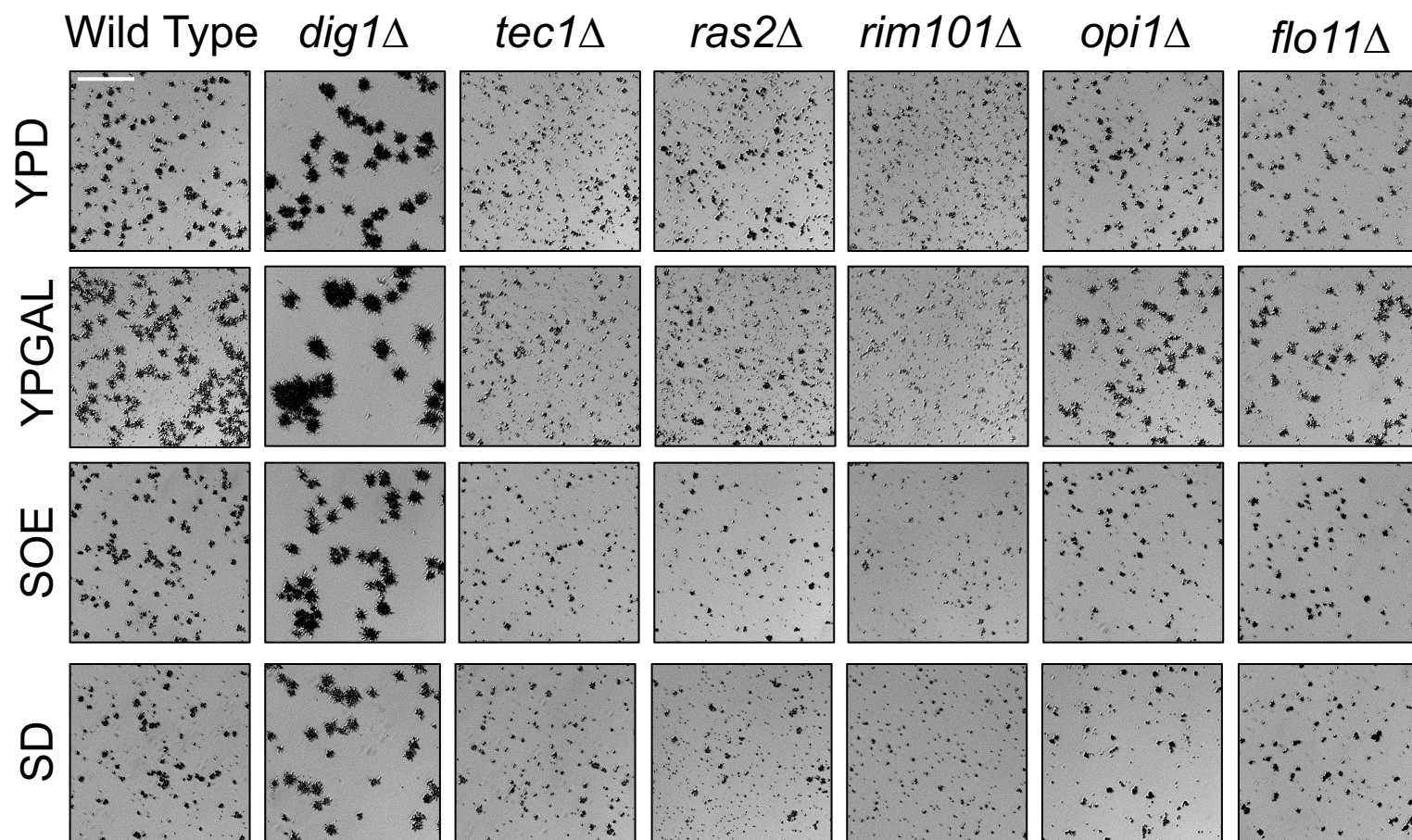**B**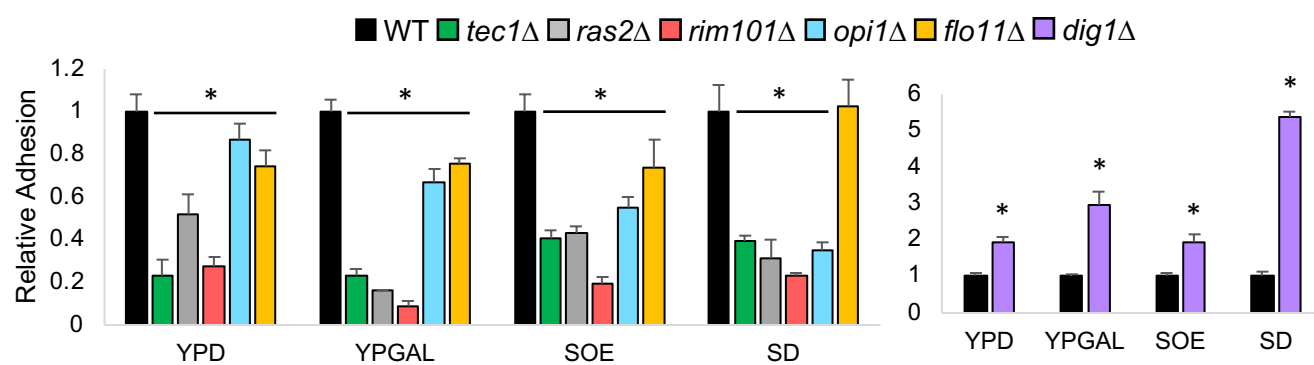

Supplement: S10 Fig — A) Cell adhesion in liquid cultures. Cells were grown in indicated media and imaged by microscopy at 5X magnification, bar, 200 μm. F) Quantification of cell clusters. Asterisk, p-value ≤ 0.05, compared to wild type. (PDF) [file pgen.1009988.s010.pdf]

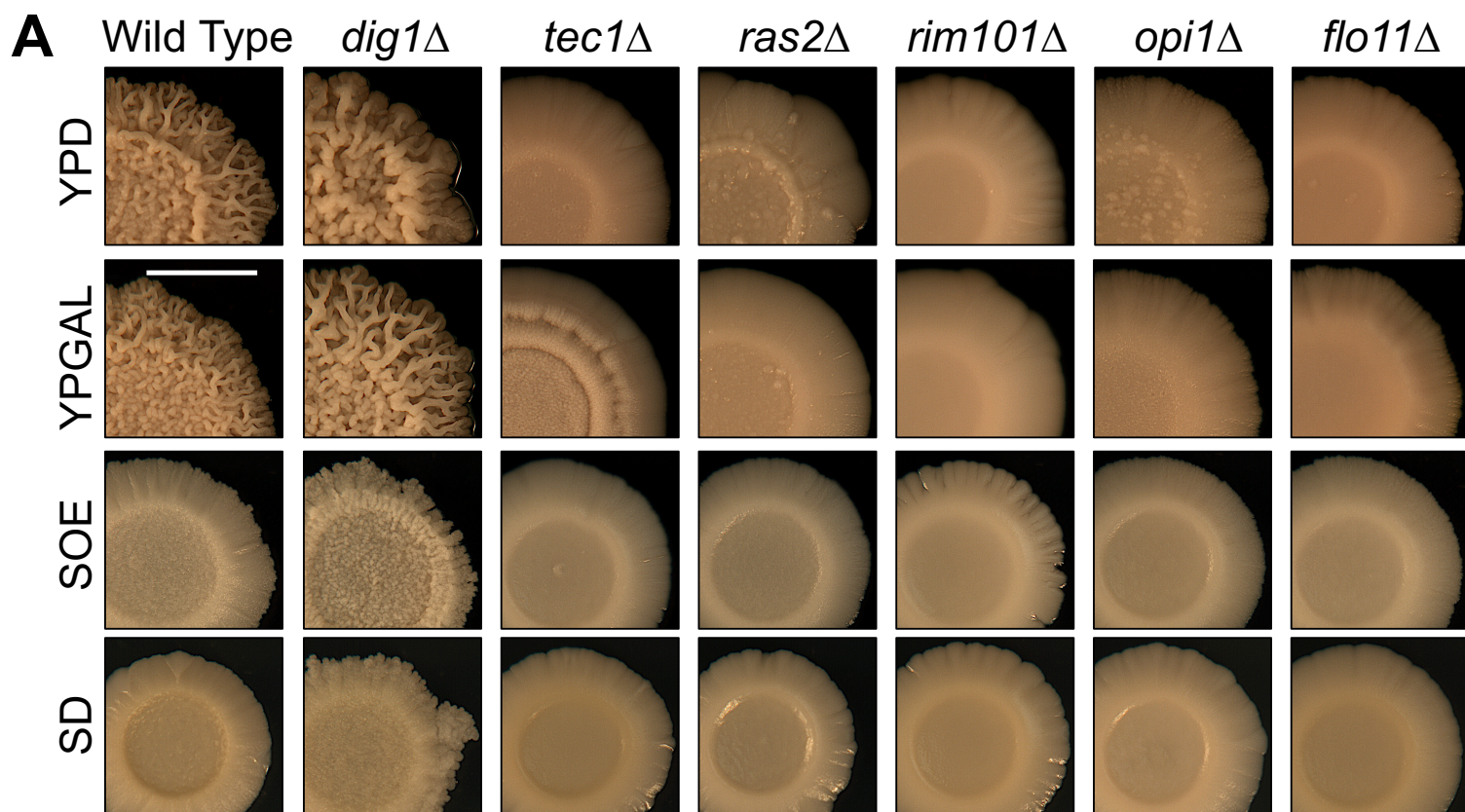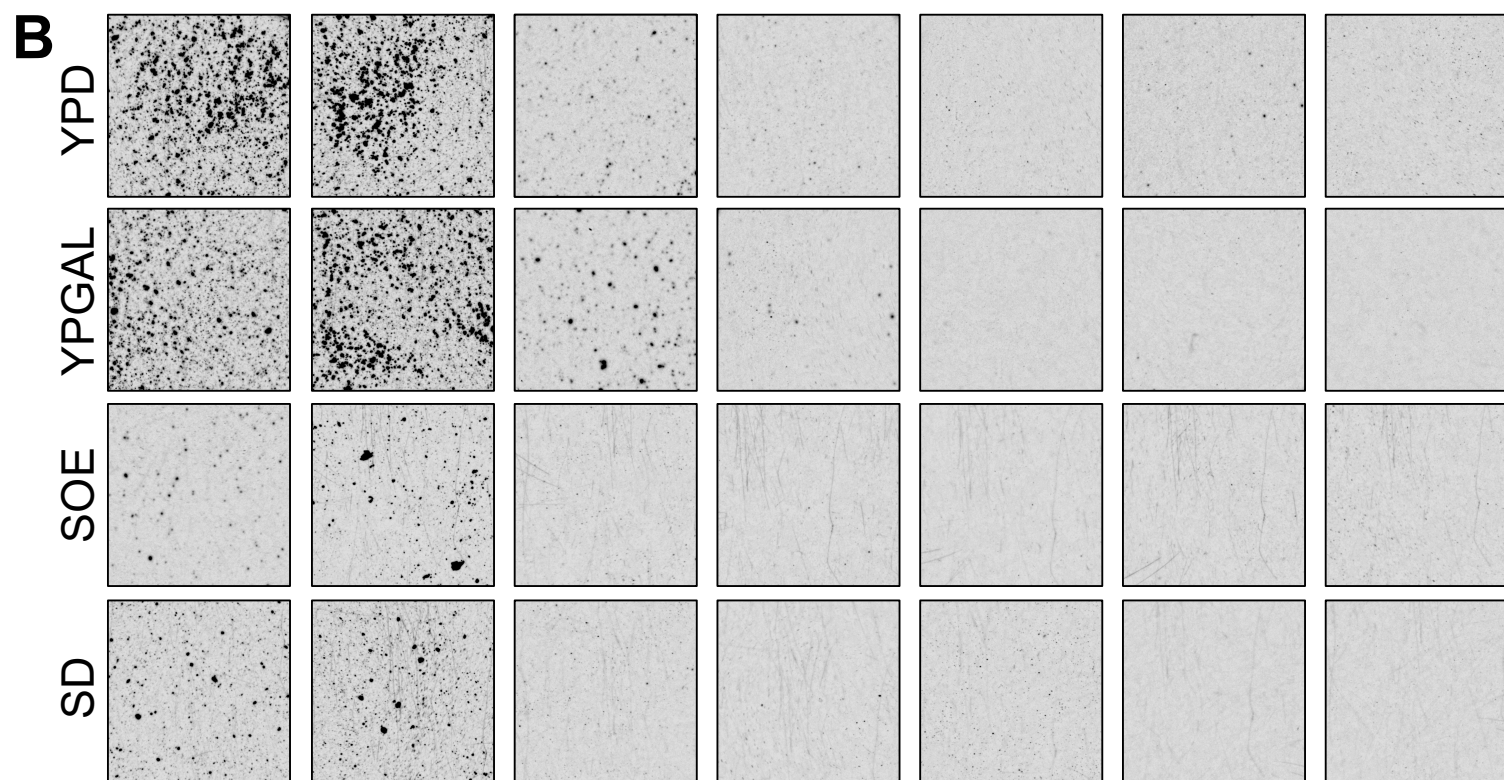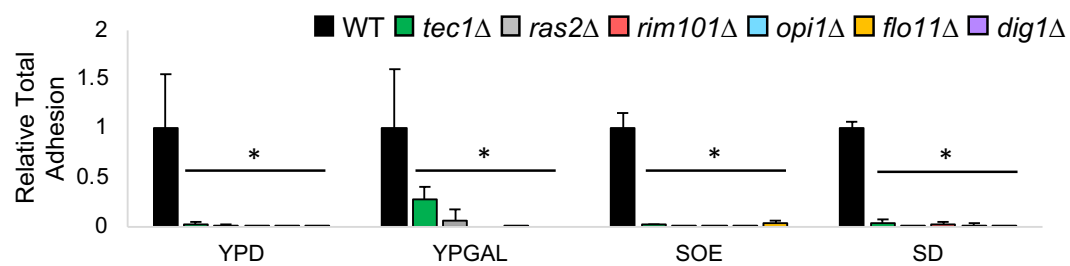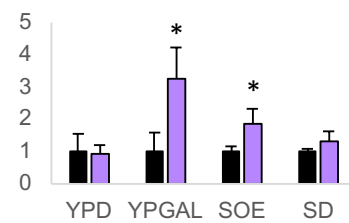

Supplement: S11 Fig — A) Complex-colony morphology, or patterning on the surface of a community of cells. The more ruffly a complex-colony morphology the more adhesion between cells. Images of colony after 7 d of growth on indicated media, bar, 0.5 cm. Wild-type cells show strong ruffling on YPD and YPGAL media and weaker but still increased complex-colony morphology compared to the flo11Δ mutant on SOE and SD media. The flo11Δ mutant exhibited a smooth pattern in all environments. All mutants tested showed reduced complex-colony morphology compared to wild type. Compared to the flo11Δ mutant the following mutants showed increased complex-colony morphology on indicated media: tec1Δ mutant on YPGAL medium; ras2Δ mutant on YPD medium; rim101Δ mutant on SOE medium; opi1Δ mutant on YPD medium; dig1Δ mutant on all media. B) Total cell adhesion within a colony. Images of adherent cells from the colony surface are seen as black particles. Bar graphs, levels of relative total adhesion compared to wild type, with wild-type values set to 1. Asterisk, P-value ≤ 0.05, compared to wild type. The flo11Δ mutant exhibited no detectable adhesion within the colony on any environment. Wild-type cells showed cell-cell adhesion on all four environments. The tec1Δ and ras2Δ mutants showed increased adhesion on YPGAL medium compared to the flo11Δ mutant. (PDF) [file pgen.1009988.s011.pdf]

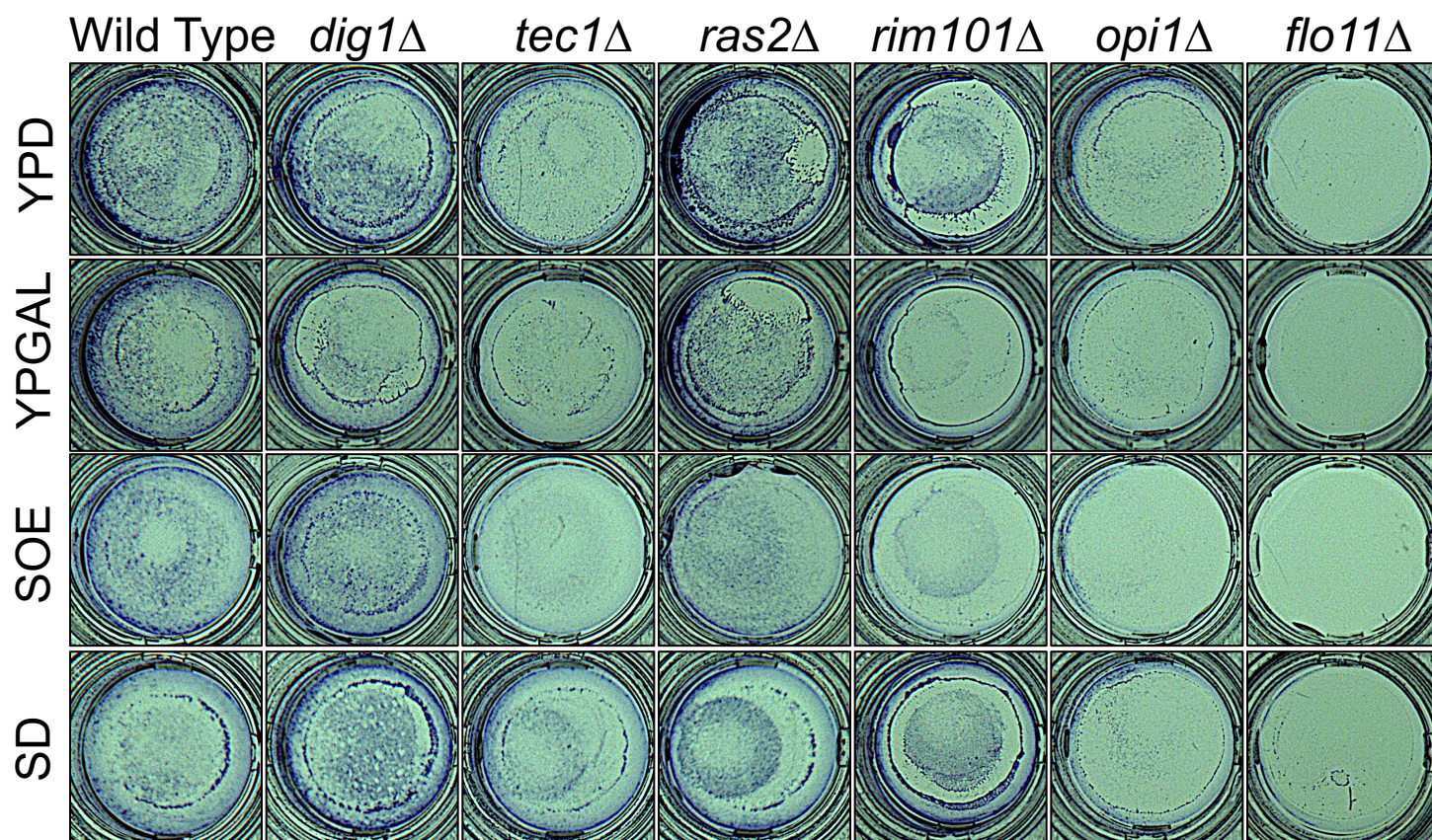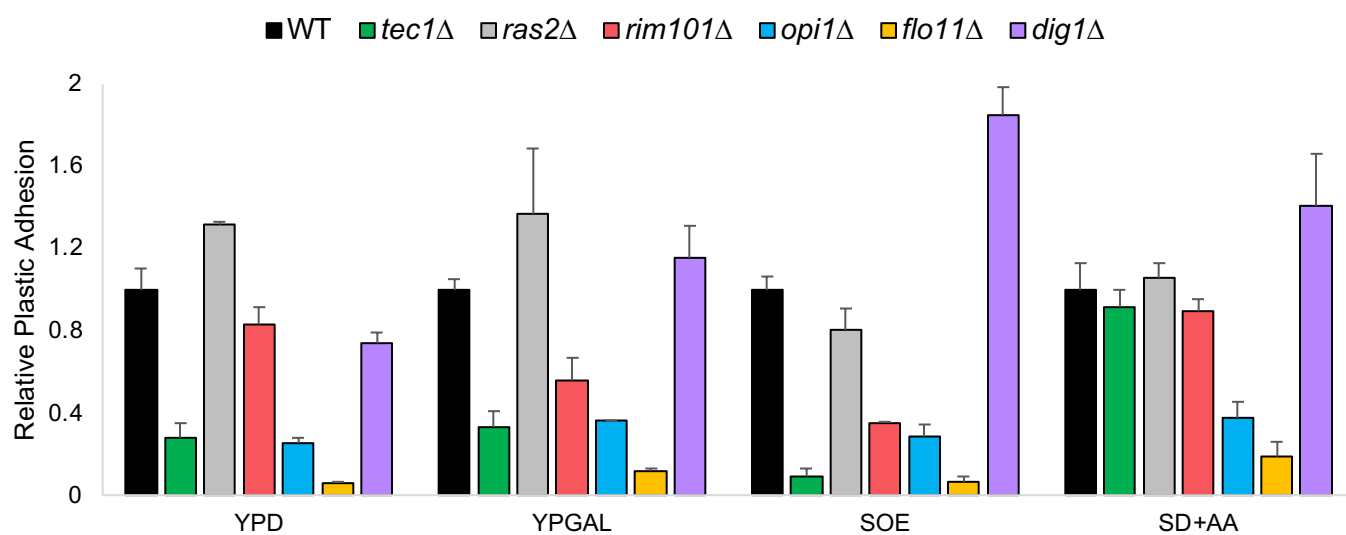

Supplement: S12 Fig — Plastic adhesion is a medically relevant phenotype because pathogenic yeasts, like Candida albicans, will adhere to medical devices and plastics in hospital settings. Images of stained cells adhering to a polystyrene plastic 96-well plate. Bar graph, quantification of relative plastic adhesion to wild type, with wild type values set to 1. (PDF) [file pgen.1009988.s012.pdf]

**A**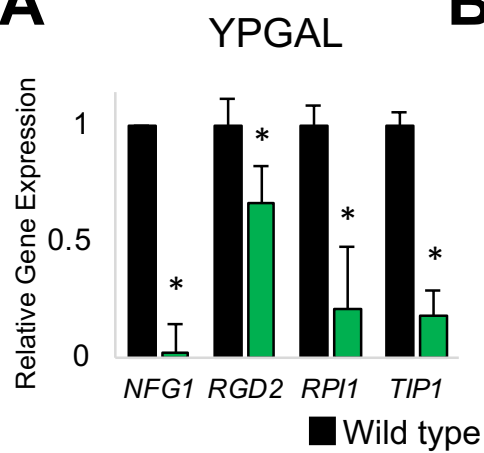**B**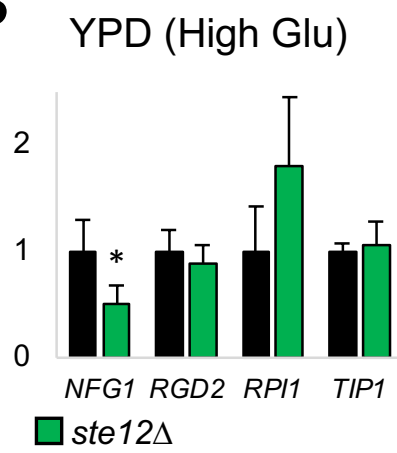**C**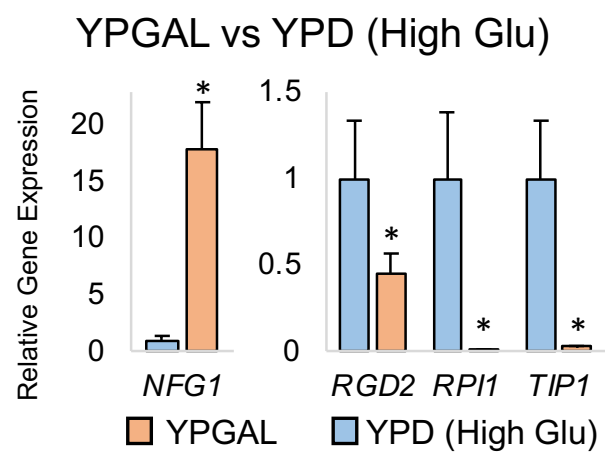

Supplement: S13 Fig — Targets of the MAPK pathway, NFG1, RGD2, RPI1, and TIP1 identified previously [100], were regulated by MAPK in one environment but not another. A) RT-qPCR analysis of mRNA levels for indicated genes between wild-type and the ste12Δ mutant (a MAPK pathway mutant equivalent to tec1Δ) in YPGAL medium. Wild-type values were normalized to ACT1 expression and set to 1. Asterisk, p-value ≤ 0.008, compared to wild type. RT-qPCR data for YPGAL comes from [100]. B) Same as panel A, except on YPD (High Glu) medium. C) These genes also show different changes in expression between the two environments. RT-qPCR analysis of mRNA levels between YPGAL and YPD (High Glu). Wild-type values were normalized to ACT1 expression. YPD (High Glu) values were set to 1. Asterisk, p-value ≤ 0.005, comparing YPGAL to YPD (High Glu). (PDF) [file pgen.1009988.s013.pdf]

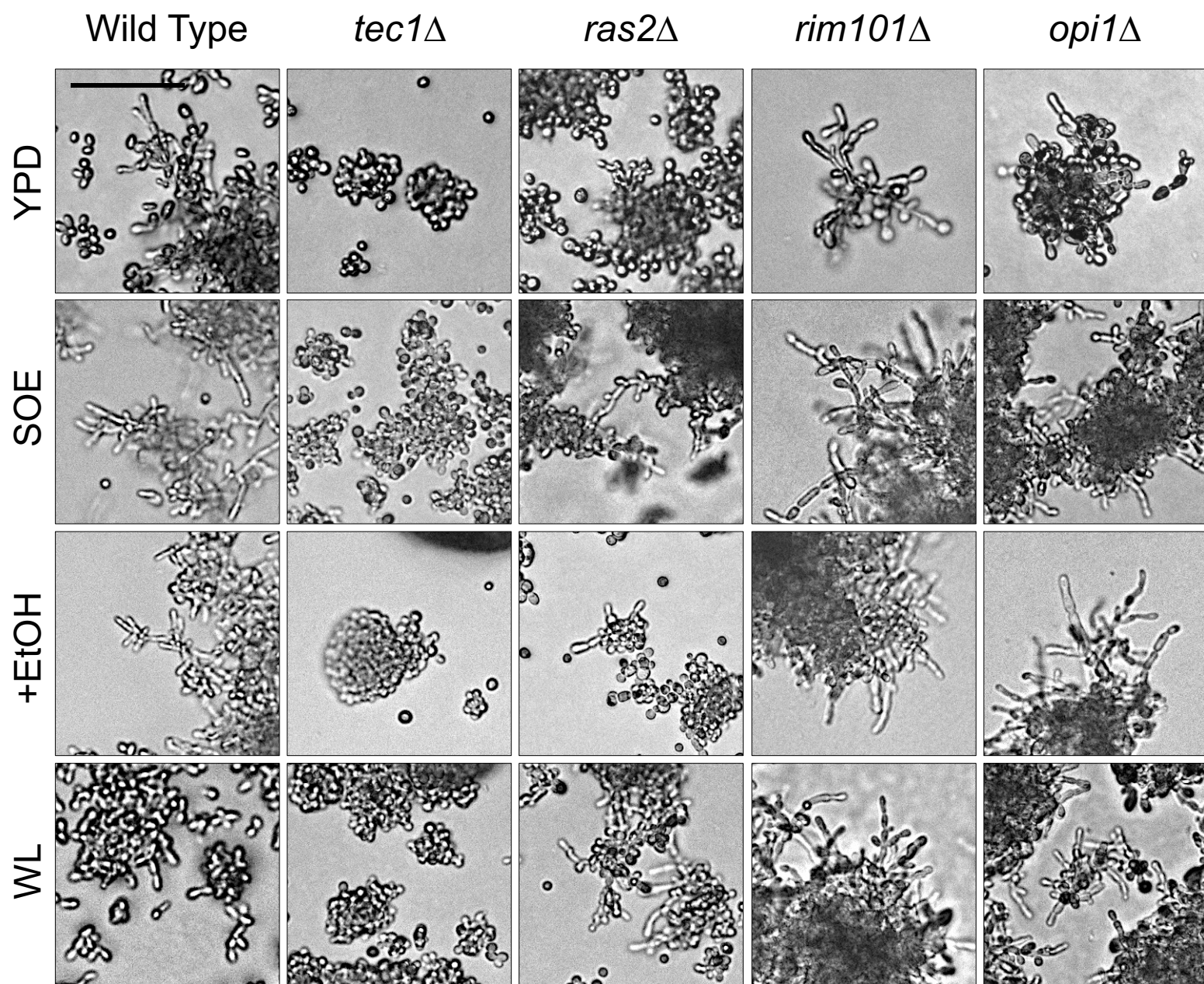

Supplement: S14 Fig — Microscopy images at 20X magnification of invasive scars on indicated media, bar, 50 μm. Each strain, except the tec1Δ mutant, showed the capability of producing a filament-like structure in each environment tested. (PDF) [file pgen.1009988.s014.pdf]

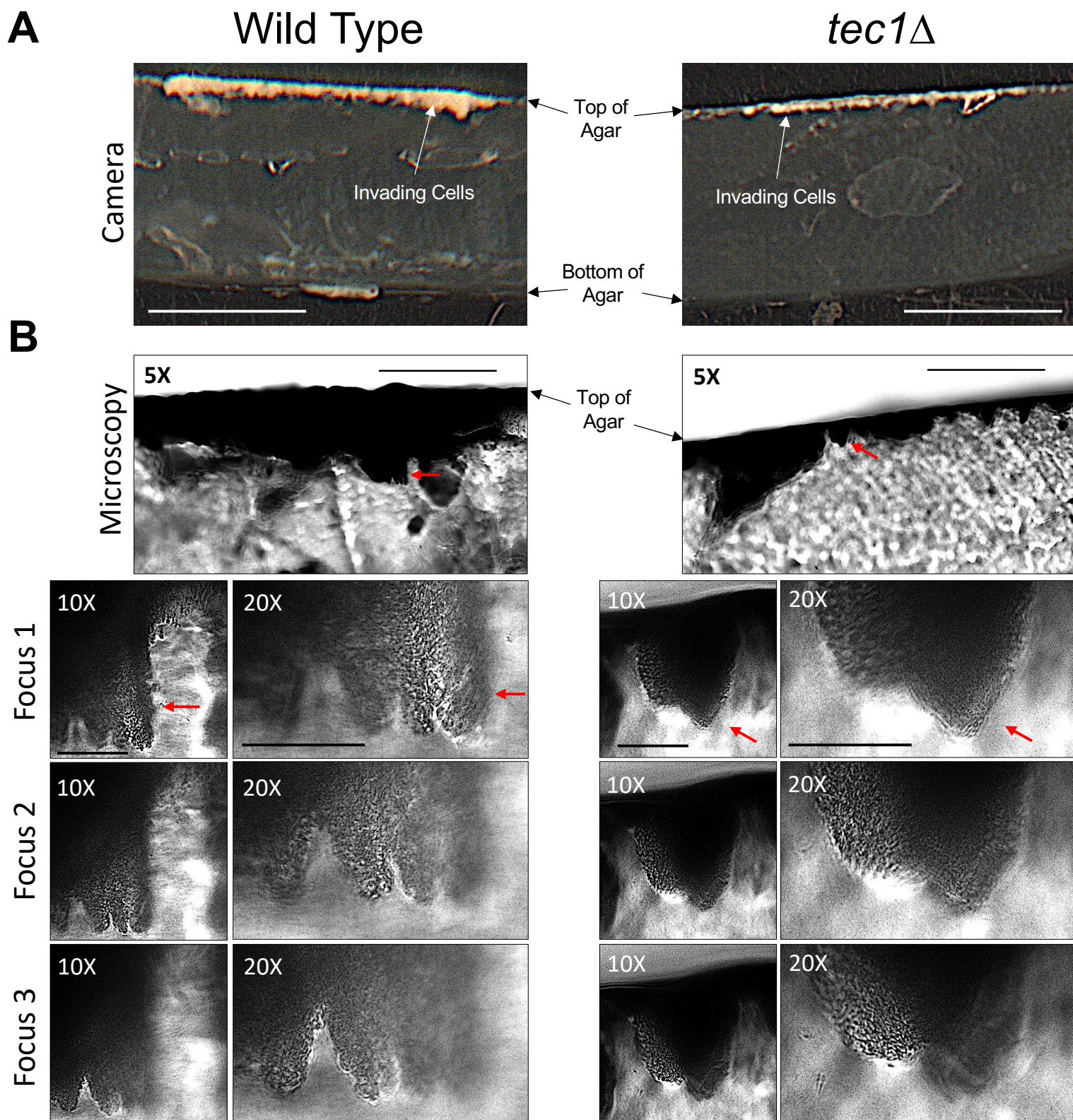

Supplement: S15 Fig — The PWA was performed on SGAL medium. A small, thin section of the invasive scar was cut and placed on its side to view the cross section. A) Colored image of invasive scar cross section by a Nikon D3000 digital camera, bar, 0.5 cm. B) Microscopy images of invasive scar cross section. 5X magnification, bar, 500 μm. Red arrows, invading cells. 10X magnification, bar, 100 μm. Red arrows, same invading cells in 5X image. 20X magnification, bar, 100 μm. Red arrows, same invading cells in 5X and 10X images. For the 10X and 20X magnification images, 3 focal planes were imaged of the same cells (Focus 1/2/3). (PDF) [file pgen.1009988.s015.pdf]

**A**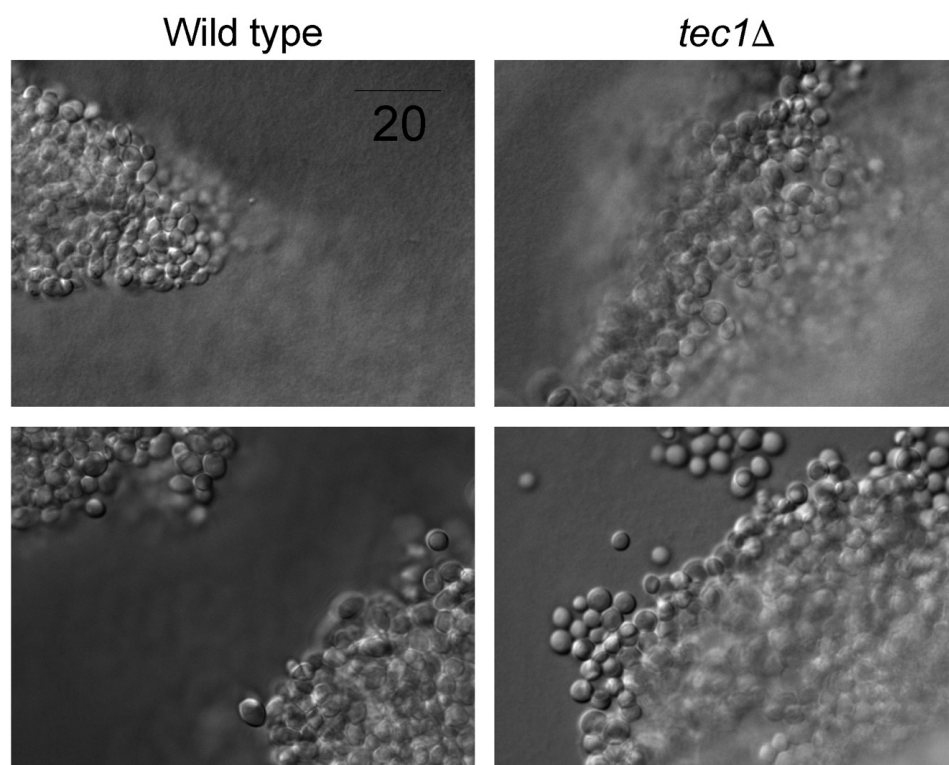**B**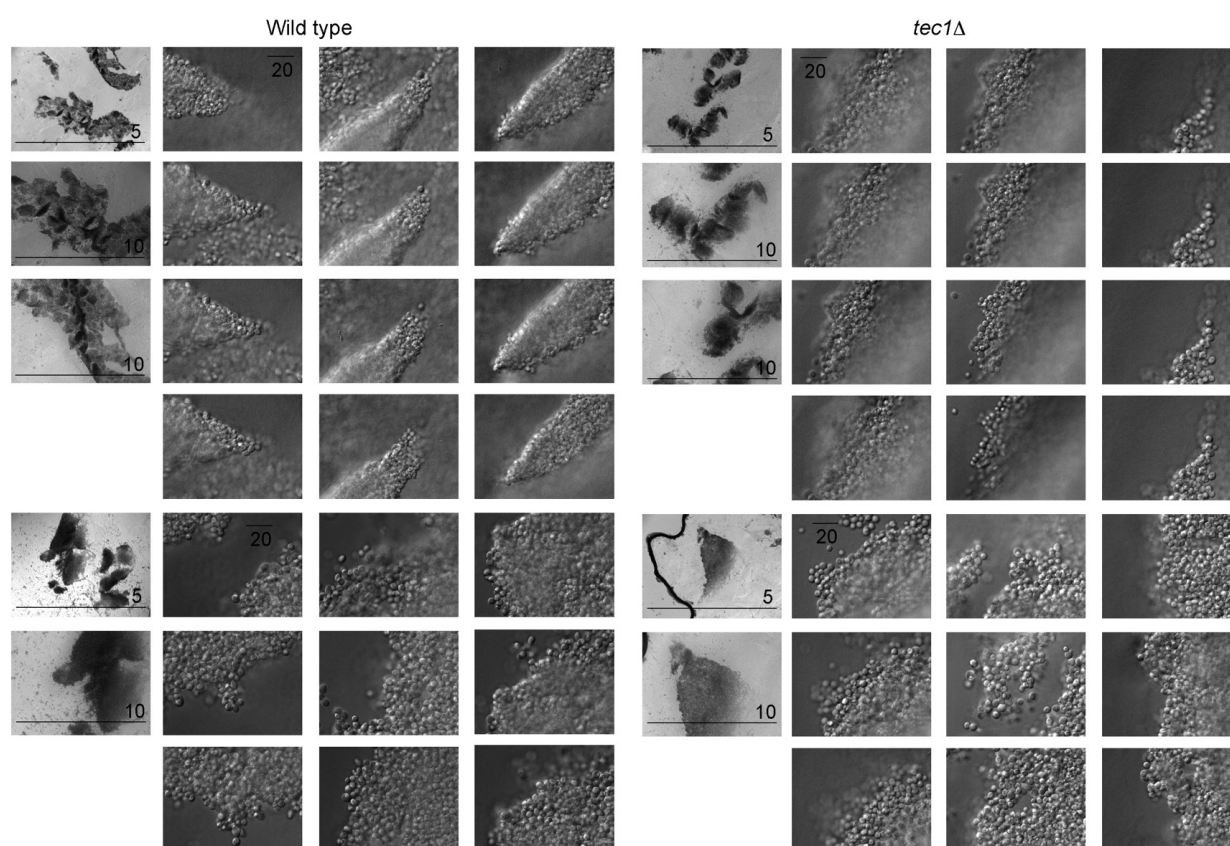

Supplement: S16 Fig — A) Examples of invaded cells for wild-type cells and the tec1D mutant. Cells were visualized by microscopy at the 100X objective. Bar, 20 microns. B) Raw data of invasive squashes of wild-type cells and the tec1D mutant. Cells were visualized by microscopy at the 5X, 10X, and 100X objectives. Bars, 5, 10, and 20 microns as indicated. Several examples are shown from different squashes. (PDF) [file pgen.1009988.s016.pdf]
