# Supplementary material for: Gene by Environment Interactions reveal new regulatory aspects of signaling network plasticity
Source: PLoS Genet. 2022 Jan 4;18(1):e1009988. doi: 10.1371/journal.pgen.1009988 (PMC8759647; doi:10.1371/journal.pgen.1009988)
Supplement: S3 Fig — A) Ranking of network components per their regulatory role of invasive growth in environments where invasive growth occurs (11 out of 12 environments, excludes +KCl). A) The average relative invasion compared to wild type (set to 1) was calculated across all environments for network mutants. Error represents standard deviation. B) Total number of environments a network mutant met a threshold of decreased invasion relative to wild type for indicated thresholds: ≤ 75%, ≤ 50%, ≤ 25%, ≤ 20%, ≤ 15%, or ≤ 10% of wild-type invasion. Each threshold was totaled independently. The pathways were ranked in order using all thresholds (# of thresholds). (PDF) [file pgen.1009988.s003.pdf]

FIG_S3

A

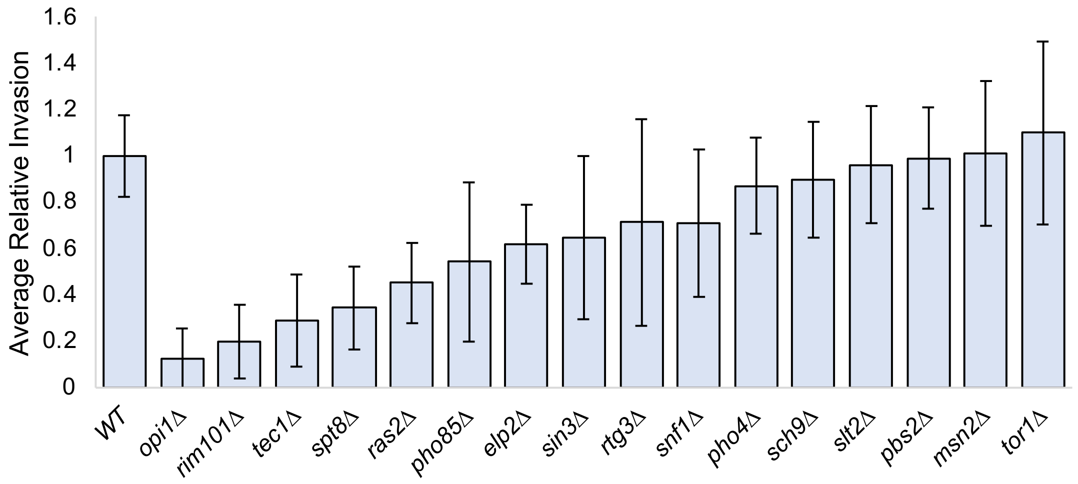

B

Number of Environments with Less Invasion than Wild Type

Network Mutants	≤ 75% Invasion	≤ 50% invasion	≤ 25% Invasion	≤ 20% Invasion	≤ 15% Invasion	≤ 10% Invasion	# of Thresholds
opi1Δ	11	11	10	10	9	4	55
rim101Δ	11	10	7	7	5	2	42
tec1Δ	11	9	7	3	3	1	34
spt8Δ	11	9	3	1	0	0	24
ras2Δ	11	6	1	1	1	1	21
pho85Δ	9	6	0	0	0	0	15
sin3Δ	8	5	1	1	0	0	15
elp2Δ	9	2	1	0	0	0	12
rtg3Δ	8	4	0	0	0	0	12
snf1Δ	6	1	1	1	1	0	10
msn2Δ	3	0	0	0	0	0	3
sch9Δ	2	1	0	0	0	0	3
tor1Δ	2	1	0	0	0	0	3
slt2Δ	2	0	0	0	0	0	2
pho4Δ	1	1	0	0	0	0	2
pbs2Δ	1	1	0	0	0	0	2
