# Supplementary material for: Gene by Environment Interactions reveal new regulatory aspects of signaling network plasticity
Source: PLoS Genet. 2022 Jan 4;18(1):e1009988. doi: 10.1371/journal.pgen.1009988 (PMC8759647; doi:10.1371/journal.pgen.1009988)
Supplement: S8 Fig — A) PWA; First column, cells before wash, second column, inverted images of scars after wash, bar, 0.5 cm. B) Levels of relative invasion to wild type, with wild type values set to 1. Asterisk, p-value ≤ 0.05, compared to wild type. C) p-values of double mutants compared to single mutants. Red highlights, p-value ≤ 0.05. (PDF) [file pgen.1009988.s008.pdf]

FIG_S8

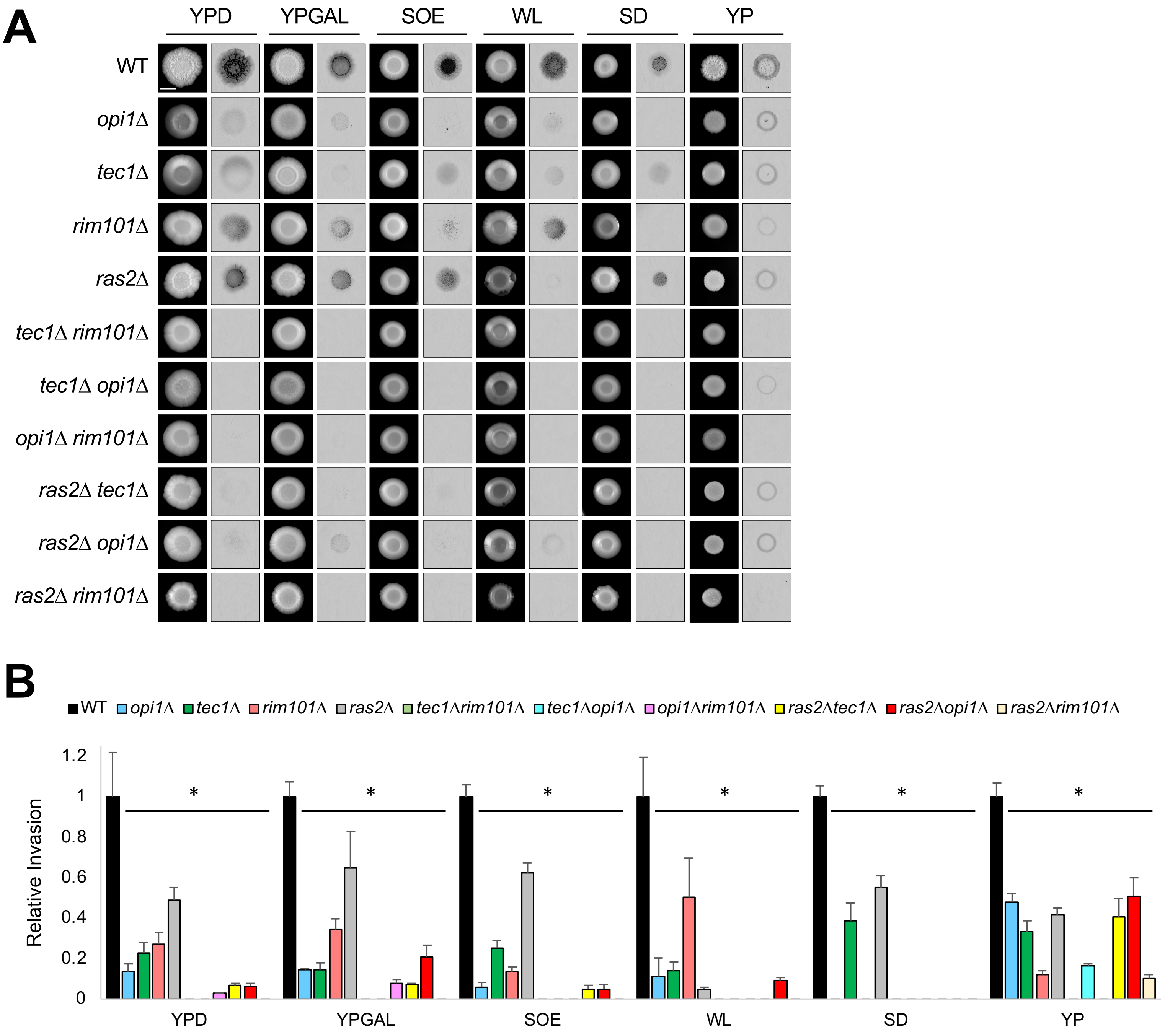

C

T-test					
Environment	Strain	opi1Δ	tec1Δ	rim101Δ	ras2Δ
YPD	tec1Δ rim101Δ		0.00173611	0.00116709	
	tec1Δ opi1Δ	0.00481924	0.00173611		
	opi1Δ rim101Δ	0.01181066		0.00182804	
	ras2Δ tec1Δ		0.00666835		0.00031607
	ras2Δ opi1Δ	0.04954016			0.00031855
	ras2Δ rim101Δ			0.00116709	0.00016783
YPGAL	tec1Δ rim101Δ		0.00193323	0.00043252	
	tec1Δ opi1Δ	1.4233E-06	0.00193323		
	opi1Δ rim101Δ	0.00293218		0.00140609	
	ras2Δ tec1Δ		0.02316249		0.00485113
	ras2Δ opi1Δ	0.13752725			0.01476348
	ras2Δ rim101Δ			0.00043252	0.00316976
SOE	tec1Δ rim101Δ		0.000305	0.00064352	
	tec1Δ opi1Δ	0.02429034	0.000305		
	opi1Δ rim101Δ	0.02429034		0.00064352	
	ras2Δ tec1Δ		0.00116568		0.00383587
	ras2Δ opi1Δ	0.23927385			0.00394495
	ras2Δ rim101Δ			0.00064352	0.00265525

T-test					
Environment	Strain	opi1Δ	tec1Δ	rim101Δ	ras2Δ
WL	tec1Δ rim101Δ		0.00491384	0.0115111	
	tec1Δ opi1Δ	0.01244042	0.00491384		
	opi1Δ rim101Δ	0.01244042		0.0115111	
	ras2Δ tec1Δ		0.00491384		0.00041574
	ras2Δ opi1Δ	0.37750188			0.00976787
	ras2Δ rim101Δ			0.0115111	0.00041574
SD	tec1Δ rim101Δ		0.00156327	N/A	
	tec1Δ opi1Δ	N/A	0.00156327		
	opi1Δ rim101Δ	N/A		N/A	
	ras2Δ tec1Δ		0.00156327		7.5466E-05
	ras2Δ opi1Δ	N/A			7.5466E-05
	ras2Δ rim101Δ			N/A	7.5466E-05
YP	tec1Δ rim101Δ		0.00035799	0.00025742	
	tec1Δ opi1Δ	0.00026958	0.00484314		
	opi1Δ rim101Δ	4.6577E-05		0.00025742	
	ras2Δ tec1Δ		0.32320036		0.82028186
	ras2Δ opi1Δ	0.68186607			0.19412493
	ras2Δ rim101Δ			0.244482	0.00012145
