# Supplementary material for: Gene by Environment Interactions reveal new regulatory aspects of signaling network plasticity
Source: PLoS Genet. 2022 Jan 4;18(1):e1009988. doi: 10.1371/journal.pgen.1009988 (PMC8759647; doi:10.1371/journal.pgen.1009988)
Supplement: S1 Table — (PDF) [file pgen.1009988.s020.pdf]

S1 Table. Media used in this study representing diverse environments.

Media*	Shorthand	Purpose
Yeast Extract, Peptone, Dextrose	YPD	Rich medium with preferred fermentable carbon source - glucose
Yeast Extract, Peptone, Galactose	YPGAL	Rich medium with non-preffered carbon source - galactose
Yeast Extract, Peptone	YP	Severe carbon stress
YPD + 1M KCl	+KCl	High osmolarity stress, which inhibits filamentous growth
Synthetic with Dextrose	SD	Synthetic medium with preferred fermentable carbon source - glucose
Synthetic with Galactose	SGAL	Synthetic medium with non-preffered carbon source - galactose
Synthetic Low Phosphate Dextrose	SLPD	Limited phosphate stress
Synthetic Low Ammonium Dextrose	SLAD	Limited nitrogen and amino acid stress
SLAD + 2% Ethanol	+EtOH	EtOH is a presumed quorum sensing molecule and has been shown to stimulate filamentous growth
Synthetic Oak Extract	SOE	Representing the natural oak tree environment yeast can be isolated in
Malt Extract	ME	Malt extract can be found in environments during beer brewing and bread baking and is part of isolation media
Wallerstein Laboratory	WL	Used to differentiatie species of yeast isolates in mixed cultures
YPD with 10% Glucose	YPD (High Glu)	YPD medium with increased glucose and osmolarity stress

* References and recipes for media in Material & Methods section
