# Supplementary material for: Gene by Environment Interactions reveal new regulatory aspects of signaling network plasticity
Source: PLoS Genet. 2022 Jan 4;18(1):e1009988. doi: 10.1371/journal.pgen.1009988 (PMC8759647; doi:10.1371/journal.pgen.1009988)
Supplement: S2 Table — (PDF) [file pgen.1009988.s021.pdf]

S2 Table. References for the cross regulation of pathway components.

Target Pathway	Target Component	Regulatory Pathway	Transcription Factor	Analysis	Reference
**MAPK**	*MSB2*	**RIM101**	Rim101p	RT-qPCR	[1]
		**OPI1**	Opi1p	RT-qPCR	[1]
		**RAS**	Flo8p	RT-qPCR	[1]
			Phd1p	CHIP	[2]
			Sok2p	CHIP	[3]
	KSS1	**OPI1**	Opi1p	Microarray	[4]
	TEC1	**RIM101**	Rim101p	Microarray	[1]
		**RAS**	Flo8p, Phd1p, Sok2p	CHIP	[2]
			--------------------------	RNA-sequencing of *ras2* Δ mutant	[5]
**RAS**	*RAS2*	**fMAPK**	Ste12p	Microarray	[6]
		**RIM101**	Rim101p	RNA-sequencing	[7]
		**OPI1**	Opi1p	Microarray	[4]
	FLO8	**fMAPK**	Tec1p, Ste12p	Microarray	[8]
				CHIP	[3]
		**OPI1**	Opi1p	Microarray	[9]
**RIM101**	*RIM101*	**RAS**	Flo8p	Microarray	[4]
			Sok2p	CHIP	[3]
	RIM8	**fMAPK**	Tec1p, Ste12p	RT-qPCR	[1]
				Microarray	[8]
**OPI1**	*OPI1*	**RIM101**	Rim101p	RNA-sequencing	[7]

References

1. Chavel CA, Dionne HM, Birkaya B, Joshi J, Cullen PJ. Multiple signals converge on a differentiation MAPK pathway. PLoS Genet. 2010;6(3):e1000883. Epub 2010/03/25. doi: 10.1371/journal.pgen.1000883. PubMed PMID: 20333241; PubMed Central PMCID: PMCPMC2841618.
2. Borneman AR, Leigh-Bell JA, Yu H, Bertone P, Gerstein M, Snyder M. Target hub proteins serve as master regulators of development in yeast. Genes Dev. 2006;20(4):435-48. Epub 2006/02/02. doi: 10.1101/gad.1389306. PubMed PMID: 16449570; PubMed Central PMCID: PMCPMC1369046.
3. Borneman AR, Zhang ZD, Rozowsky J, Seringhaus MR, Gerstein M, Snyder M. Transcription factor binding site identification in yeast: a comparison of high-density oligonucleotide and PCR-based microarray platforms. Funct Integr Genomics. 2007;7(4):335-45. Epub 2007/07/20. doi: 10.1007/s10142-007-0054-7. PubMed PMID: 17638031.
4. Reimand J, Vaquerizas JM, Todd AE, Vilo J, Luscombe NM. Comprehensive reanalysis of transcription factor knockout expression data in Saccharomyces cerevisiae reveals many new targets. Nucleic Acids Res. 2010;38(14):4768-77. Epub 2010/04/14. doi: 10.1093/nar/gkq232. PubMed PMID: 20385592; PubMed Central PMCID: PMCPMC2919724.
5. Adhikari H, Cullen PJ. Metabolic respiration induces AMPK- and Ire1p-dependent activation of the p38-Type HOG MAPK pathway. PLoS Genet. 2014;10(10):e1004734. Epub 2014/10/31. doi: 10.1371/journal.pgen.1004734. PubMed PMID: 25356552; PubMed Central PMCID: PMCPMC4214603.
6. Chua G, Morris QD, Sopko R, Robinson MD, Ryan O, Chan ET, et al. Identifying transcription factor functions and targets by phenotypic activation. Proc Natl Acad Sci U S A. 2006;103(32):12045-50. Epub 2006/08/02. doi: 10.1073/pnas.0605140103. PubMed PMID: 16880382; PubMed Central PMCID: PMCPMC1567694.
7. Read T, Richmond PA, Dowell RD. A trans-acting Variant within the Transcription Factor RIM101 Interacts with Genetic Background to Determine its Regulatory Capacity. PLoS Genet. 2016;12(1):e1005746. Epub 2016/01/12. doi: 10.1371/journal.pgen.1005746. PubMed PMID: 26751950; PubMed Central PMCID: PMCPMC4709078.
8. Madhani HD, Galitski T, Lander ES, Fink GR. Effectors of a developmental mitogen-activated protein kinase cascade revealed by expression signatures of signaling mutants. Proc Natl Acad Sci U S A. 1999;96(22):12530-5. Epub 1999/10/27. doi: 10.1073/pnas.96.22.12530. PubMed PMID: 10535956; PubMed Central PMCID: PMCPMC22972.
9. Hickman MJ, Petti AA, Ho-Shing O, Silverman SJ, McIsaac RS, Lee TA, et al. Coordinated regulation of sulfur and phospholipid metabolism reflects the importance of methylation in the growth of yeast. Mol Biol Cell. 2011;22(21):4192-204. Epub 2011/09/09. doi: 10.1091/mbc.E11-05-0467. PubMed PMID: 21900497; PubMed Central PMCID: PMCPMC3204079.
