# Supplementary material for: Gene by Environment Interactions reveal new regulatory aspects of signaling network plasticity
Source: PLoS Genet. 2022 Jan 4;18(1):e1009988. doi: 10.1371/journal.pgen.1009988 (PMC8759647; doi:10.1371/journal.pgen.1009988)
Supplement: S3 Table — (PDF) [file pgen.1009988.s022.pdf]

S3 Table. Yeast strains used in this study.

Strain	Description	Reference
PC313	*MAT***a** *SY3089 ura3-52*	[1]
PC368	*MAT***a** *SY3089 ura3-52 pbs2::URA3*	[2]
PC538	*MAT***a** *SY3089 ste4 FUS1-lacZ FUS1-HIS3 ura3-52*	[3]
PC539	*MAT***a** *SY3089 ste4 FUS1-lacZ FUS1-HIS3 ura3-52 ste12::URA3*	[4]
PC551	*MAT***a** *SY3089 ste4 FUS1-lacZ FUS1-HIS3 ura3-52 pea2::URA3*	[2]
PC560	*MAT***a** *SY3089 ste4 FUS1-lacZ FUS1-HIS3 ura3-52 snf1::URA3*	[2]
PC562	*MAT***a** *SY3089 ste4 FUS1-lacZ FUS1-HIS3 ura3-52 ras2::URA3*	[2]
PC563	*MAT***a** *SY3089 ste4 FUS1-lacZ FUS1-HIS3 ura3-52 bud8::KlURA3*	[5]
PC569	*MAT***a** *SY3089 ste4 FUS1-lacZ FUS1-HIS3 ura3-52 tec1::URA3*	[2]
PC654	*MAT***a** *SY3089 ste4 FUS1-lacZ FUS1-HIS3 ura3-52 pho85::URA3*	[6]
PC1029	*MAT***a** *SY3089 ste4 FUS1-lacZ FUS1-HIS3 ura3-52 flo11::KanMX6*	[7]
PC2110	*MAT***a** *SY3089 ste4 FUS1-lacZ FUS1-HIS3 ura3-52 leu2::HYG fus1lacz::NAT pbs2::GENT*	[4]
PC2847	*MAT***a** *SY3089 ste4 FUS1-lacZ FUS1-HIS3 ura3-52 FLO11-HA at 1000aa opi1::URA3*	[6]
PC2953	*MAT***a** *SY3089 ste4 FUS1-lacZ FUS1-HIS3 ura3-52 Msb2-HA at 500aa rim101::URA3*	[6]
PC2976	*MAT***a** *SY3089 ste4 FUS1-lacZ FUS1-HIS3 ura3-52 Msb2-HA at 500aa elp2::URA3*	[8]
PC3030	*MAT***a** *SY3089 ste4 FUS1-lacZ FUS1-HIS3 ura3-52 Msb2-HA at 500aa sin3::NAT*	[6]
PC3039	*MAT***a** *SY3089 ste4 FUS1-lacZ FUS1-HIS3 ura3-52 Msb2-HA at 500aa dig1::KlURA3*	[6]
PC3188	*MAT***a** *SY3089 ste4 FUS1-lacZ FUS1-HIS3 ura3-52 Msb2-HA at 500aa slt2::KlURA3*	[6]
PC3353	*MAT***a** *SY3089 ura3-52 STE12-HA sin3::NAT*	[6]
PC3654	*MAT***a** *SY3089 ste4 FUS1-lacZ FUS1-HIS3 ura3-52 Msb2-HA at 500aa tor1::NAT*	[6]
PC3698	*MAT***a** *SY3089 ura3-52 leu2 rtg3::NAT*	[6]
PC4008	*MAT***a** *SY3089 ste4 FUS1-lacZ FUS1-HIS3 ura3-52 spt8::URA3*	[9]
PC5115	*MAT***a** *SY3089 ste4 FUS1-lacZ FUS1-HIS3 ura3-52 pho4::NAT*	[9]
PC5864	*MAT***a** *SY3089 ste4 FUS1-lacZ FUS1-HIS3 ura3-52 sch9::URA3*	[9]
PC6094	*MAT***a** *SY3089 ste4 FUS1-lacZ FUS1-HIS3 ura3-52 msn2::URA3*	[9]
PC6222	*MAT***a** *SY3089 ura3-52 ras2::HYG*	[9]
PC7673	*MAT***a** *SY3089 ura3-52 rim101::NAT*	This study
PC7674	*MAT***a** *SY3089 ura3-52 opi1::NAT*	This study
PC7675	*MAT***a** *SY3089 ura3-52 tec1::NAT*	This study
PC7676	*MAT***a** *SY3089 ura3-52 dig1::NAT*	This study
PC7677	*MAT***a** *SY3089 ura3-52 rtg3::NAT*	This study
PC7678	*MAT***a** *SY3089 ste4 FUS1-lacZ FUS1-HIS3 ura3-52 ras2::URA3 tec1::NAT*	This study
PC7679	*MAT***a** *SY3089 ste4 FUS1-lacZ FUS1-HIS3 ura3-52 tec1::URA3 opi1::NAT*	This study
PC7680	*MAT***a** *SY3089 ste4 FUS1-lacZ FUS1-HIS3 ura3-52 tec1::URA3 rim101::NAT*	This study
PC7681	*MAT***a** *SY3089 ste4 FUS1-lacZ FUS1-HIS3 ura3-52 FLO11-HA at 1000aa opi1::URA3 rim101::NAT*	This study
PC7689	*MAT***a** *SY3089 ste4 FUS1-lacZ FUS1-HIS3 ura3-52 ras2::URA3 opi1::NAT*	This study
PC7690	*MAT***a** *SY3089 ste4 FUS1-lacZ FUS1-HIS3 ura3-52 ras2::URA3 rim101::NAT*	This study

References

1. Liu H, Styles CA, Fink GR. Elements of the yeast pheromone response pathway required for filamentous growth of diploids. Science. 1993;262(5140):1741-4. Epub 1993/12/10. doi: 10.1126/science.8259520. PubMed PMID: 8259520.
2. Cullen PJ, Sprague GF, Jr. Glucose depletion causes haploid invasive growth in yeast. Proc Natl Acad Sci U S A. 2000;97(25):13619-24. Epub 2000/11/30. doi: 10.1073/pnas.240345197. PubMed PMID: 11095711; PubMed Central PMCID: PMCPMC17625.
3. Cullen PJ, Sabbagh W, Jr., Graham E, Irick MM, van Olden EK, Neal C, et al. A signaling mucin at the head of the Cdc42- and MAPK-dependent filamentous growth pathway in yeast. Genes Dev. 2004;18(14):1695-708. Epub 2004/07/17. doi: 10.1101/gad.1178604. PubMed PMID: 15256499; PubMed Central PMCID: PMCPMC478191.
4. Pitoniak A, Birkaya B, Dionne HM, Vadaie N, Cullen PJ. The signaling mucins Msb2 and Hkr1 differentially regulate the filamentation mitogen-activated protein kinase pathway and contribute to a multimodal response. Mol Biol Cell. 2009;20(13):3101-14. Epub 2009/05/15. doi: 10.1091/mbc.E08-07-0760. PubMed PMID: 19439450; PubMed Central PMCID: PMCPMC2704161.
5. Cullen PJ, Sprague GF, Jr. The roles of bud-site-selection proteins during haploid invasive growth in yeast. Mol Biol Cell. 2002;13(9):2990-3004. Epub 2002/09/11. doi: 10.1091/mbc.e02-03-0151. PubMed PMID: 12221111; PubMed Central PMCID: PMCPMC124138.
6. Chavel CA, Dionne HM, Birkaya B, Joshi J, Cullen PJ. Multiple signals converge on a differentiation MAPK pathway. PLoS Genet. 2010;6(3):e1000883. Epub 2010/03/25. doi: 10.1371/journal.pgen.1000883. PubMed PMID: 20333241; PubMed Central PMCID: PMCPMC2841618.
7. Karunanithi S, Vadaie N, Chavel CA, Birkaya B, Joshi J, Grell L, et al. Shedding of the mucin-like flocculin Flo11p reveals a new aspect of fungal adhesion regulation. Curr Biol. 2010;20(15):1389-95. Epub 2010/07/14. doi: 10.1016/j.cub.2010.06.033. PubMed PMID: 20619652; PubMed Central PMCID: PMCPMC2918736.
8. Abdullah U, Cullen PJ. The tRNA modification complex elongator regulates the Cdc42-dependent mitogen-activated protein kinase pathway that controls filamentous growth in yeast. Eukaryot Cell. 2009;8(9):1362-72. Epub 2009/07/28. doi: 10.1128/EC.00015-09. PubMed PMID: 19633267; PubMed Central PMCID: PMCPMC2747823.
9. Chavel CA, Caccamise LM, Li B, Cullen PJ. Global regulation of a differentiation MAPK pathway in yeast. Genetics. 2014;198(3):1309-28. Epub 2014/09/06. doi: 10.1534/genetics.114.168252. PubMed PMID: 25189875; PubMed Central PMCID: PMCPMC4224168.
