# Supplementary material for: Gene by Environment Interactions reveal new regulatory aspects of signaling network plasticity
Source: PLoS Genet. 2022 Jan 4;18(1):e1009988. doi: 10.1371/journal.pgen.1009988 (PMC8759647; doi:10.1371/journal.pgen.1009988)
Supplement: S4 Table — (PDF) [file pgen.1009988.s023.pdf]

S4 Table. RT-qPCR primers used in this study.

Gene	Forward Primer 5'-3'	Reverse Primer 5'-3'	Reference
ACT1	GGCTTCTTTGACTACCTTCCAACA	GATGGACCACTTTCGTCGTATTC	[1]
TIP1	TCTGAAATCGCTGCTGCTCT	GGCAGAGGATGTAGCTTCGG	[2]
NFG1	CACCACCTCTTCCAGCATTA	CGCTGGAAGTGACTTTTGGT	[2]
RPI1	TCGAACTCCAACTCCAACTC	CTTGCAGACGAGGAGGATGA	[2]
RGD2	ATGTGGCCAGGGAAAAGGAT	AACAGCTGAAGCGTCTCCTT	[2]
FLO11	CACTTTTGAAGTTTATGCCACACAAG	CTTGCATATTGAGCGGCACTAC	[3]
SFG1	TAAAGAAGCACTGGAGCTCC	GTGGCACATCACTGTTGGAT	[2]

References

1. Chow J, Starr I, Jamalzadeh S, Muniz O, Kumar A, Gokcumen O, et al. Filamentation Regulatory Pathways Control Adhesion-Dependent Surface Responses in Yeast. Genetics. 2019;212(3):667-90. Epub 2019/05/06. doi: 10.1534/genetics.119.302004. PubMed PMID: 31053593; PubMed Central PMCID: PMCPMC6614897.
2. Vandermeulen MD, Cullen PJ. New Aspects of Invasive Growth Regulation Identified by Functional Profiling of MAPK Pathway Targets in Saccharomyces cerevisiae. Genetics. 2020;216(1):95-116. Epub 2020/07/16. doi: 10.1534/genetics.120.303369. PubMed PMID: 32665277; PubMed Central PMCID: PMCPMC7463291.
3. Chen H, Fink GR. Feedback control of morphogenesis in fungi by aromatic alcohols. Genes Dev. 2006;20(9):1150-61. Epub 2006/04/19. doi: 10.1101/gad.1411806. PubMed PMID: 16618799; PubMed Central PMCID: PMCPMC1472474.
