# Supplementary material for: Gene by Environment Interactions reveal new regulatory aspects of signaling network plasticity
Source: PLoS Genet. 2022 Jan 4;18(1):e1009988. doi: 10.1371/journal.pgen.1009988 (PMC8759647; doi:10.1371/journal.pgen.1009988)
Supplement: Attachment — Submitted filename: Response to reviewers.pdf [file pgen.1009988.s024.pdf]

Response to Reviewer #1

Reviewer #1: This manuscript by Vandermeulen and Cullen takes advantage of the invasive growth phenotype in budding yeast to quantify gene-environment interactions and how phenotypic plasticity can be mediated through condition-specific signaling. The authors examined mutants lacking main regulators of invasive growth across multiple environments, which allowed them to determine their relative importance under different conditions. In addition to the pathways known to play major roles in regulating invasive growth, the authors found that the transcriptional regulator Opi1 plays an even larger role under most conditions. The authors tested various double mutants under different conditions, which revealed that pathways can act additively, redundantly, or even opposingly depending on the environment. Finally, the authors measured in mutants the gene expression of target genes ultimately responsible for different aspects of invasive growth, ultimately allowing them to begin to connect different pathways to expression output.

Altogether, I think this manuscript rather thoroughly describes the diverse ways that different signaling pathways can interact leading to both regulatory and organismal phenotypic plasticity. I do have some comments and suggestions, largely to clarify for the reader.

Thanks very much to the reviewer for their helpful suggestions. We have thought carefully about the reviewer's suggestions and have incorporated most of the suggested changes into the manuscript, which has improved the paper and made the conclusions clearer.

1) I think it would be helpful to have the first figure show at least the abbreviated major pathways (Rim101, Pka/Ras, MAPK) and how they connect to major output genes and cellular phenotypes relevant to invasive growth (FLO11 - adhesion, BUD8 - distal budding, PEA2 - elongation).

We have added the following model to Figure 1 and updated the figure legend.

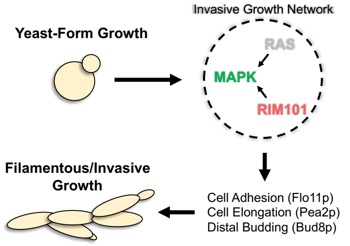

We have made changes to refer to the model in the INTRODUCTION as follows:

- "…that can penetrate into substrates (i.e. invasive growth [64], **Fig. 1A**)…"
- "…and the **RIM101** pathway [67, 81, 82], which responds to pH (**Fig. 1A**)…"
- "…Most regulatory pathways co-regulate a common set of target genes [82-86]~~, especially the gene encoding the main adhesion molecule, *FLO11* [78]~~. Key transcription factors regulate each other's expression [83], and several pathways regulate each others' activity [77, 87-89] (**Fig. 1A**, e.g. **RAS** and **RIM101** regulate **MAPK**), including at the levels of the protein kinases that comprise the pathways [90]. The filamentous/invasive growth network induces three major effector phenotypes during filamentous growth: an increase in cell-to-cell adhesion, a switch to distal-unipolar budding (from axial budding in haploids

to promote growth away from other cells), and an increase in cell length [63, 64, 91] (**Fig. 1A**)…"

2) Please consider changing the color scheme for red/green heat maps for the sake of any colorblind colleagues (blue/red should work).

We agree and have changed the color of the heat maps. The figure legends have been adjusted accordingly.

3) While I appreciated how the authors used the ranking of mutational effects to determine which pathways played the largest roles under different environments, the authors may also consider hierarchical clustering by both mutant and environment (or just calculating correlation coefficients). In particular, clustering by environment may help determine which signals are being used by which pathways (e.g. nitrogen level vs. carbon source), and it may help to determine the environmental trigger that causes certain mutants to no longer have changes in reaction norms.

We agree and have re-examined the data by statistical tests that correct p-values for multiple comparisons, and we have organized the data in different ways to show off similar patterns. Due to comments from another reviewer, we have removed the reaction norm data, which was considered distracting. This has had the effect of streamlining and focusing the paper on the main points.

4) Minor point: how was the order of mutants determined in Figure 1--it's not exactly the same as S3.

The order of the mutants in Figure 1 was based on their ranking in YPD alone (versus their average across all conditions in *Figure S3*). This analysis highlights how the traditional laboratory medium does not reflect the overall ranking from GEI. We have updated the manuscript to reflect this point on P10-P11: "Both methods showed a different overall ranking of the regulators compared to the order seen in YPD (compare the order in **Fig. 1D** to *Fig. S3*)."

5) This may be coincidental, but the opi1 mutant seems to be the phenotypic mirror image of dig1. Reynolds 2020 speculates that Opi1 is a repressor that is indirectly causing FLO11 activation. Could it be that Opi1 is a repressor for Dig1? There may be some transcriptomic datasets that could help answer that question.

According to *SGD* and YEASTRACT, Opi1 does not bind to the *DIG1* promoter. Nevertheless, this is an interesting possibility that we will keep in mind as we continue to study pathway relationships in the network.

6) For the double mutant analysis, lack of significance in a t-test is not really the same as testing for equivalency, and it may be inherently difficult with the invasive growth assay to determine non-additivity for mutants with already major defects. I think the interpretation of interacting pathways like Ras should be toned down unless there is other evidence for cross talk. Otherwise, I think testing expression of reporter genes like FLO11 in single and double mutants may be more

sensitive for identifying conditions where pathways are acting in parallel or are interacting.

We have toned down this conclusion in the revised manuscript as follows:

On P17 we made the following changes: "…This ~~demonstrates~~ may suggest that a pathway in the network can make an additive contribution in one environment (**Fig. 3C**, **RAS** on YPD), a redundant or similar contribution to one pathway in another environment (**Fig. 3C**, **RAS** on YPGAL), and a redundant or similar contribution to more than one pathway in a third environment (**Fig. 3C**, **RAS** on YP). In environments where double mutants invade, it can be inferred that at least one additional pathway is required. ~~Collectively,~~ These observations are consistent with the idea that pathways that regulate invasive growth can play additive or redundant roles depending on the environment, which we refer to as conditional redundancy…"

Evidence for conditional regulation of MAPK by the RAS pathway also comes from examination of the *FRE-lacZ* reporter in the *ras2* mutant under different conditions. We now mention these results on P20-P21 in the following sentence: "Furthermore, conditional cross-pathway regulation may account for how conditional redundancy occurs between the **RAS** and **MAPK** pathways (**Fig. 3C**)."

7) I'm misunderstanding something in Figure 4 comparing "A" and "C." The order of pathway activity in "C" does not match the order in "A." Likewise, not sure where the order in "D" for invasive growth is coming from.

We have made adjustments to the figure to improve clarity:

1. We have color coded the environments in 4A to match 4C and 4D.
2. We removed the additional invasive growth conditions in panel 4D so all conditions are the same for all panels in Figure 4.
3. We combined panels C and D into one figure and moved the key down to the bottom of the panel.
4. We added labels for "Min MAPK" and "Max MAPK"

We have also made the following changes in this section (P19-P20) to improve clarity: "…The levels of the reporter in cells lacking a positive regulator of the **MAPK** pathway ~~the *tec1*Δ mutant~~ defined a lower boundary for **MAPK** pathway activity (**Fig. 4B**, *tec1*Δ, green). Reporter levels in cells lacking a negative regulator of the **MAPK** pathway, Dig1p [147-150], defined an upper boundary (**Fig. 4B**, *dig1*Δ, purple). **MAPK** pathway activity was closer to the lower boundary in ~~most~~ the environments tested (**Fig. 4C**, **MAPK** pathway activity). ~~Although the environment that triggers maximal invasive growth may have yet to be identified, it is clear that the **MAPK** pathway is not maximally activated in environments in which cells undergo robust invasive growth. Similarly,~~ Like **MAPK** pathway activity, ~~to~~ **MAPK**-dependent invasive growth in wild-type cells ~~invasion~~ was closer to the lower boundary (represented by the hypoinvasive *tec1*Δ mutant) compared to the upper boundary (represented by the hyperinvasive *dig1*Δ mutant [*Fig. S1, Fig. S2B*]) in most environments tested, based on examination of invasive growth across conditions (**Fig. 4C**, Invasive Growth) and over time (**Fig. 4D**, *Fig. S7E*). One exception was on YP media, where invasive growth, but not ~~was similar to~~ **MAPK** pathway activity, ~~for most environments (**Fig. 4D**, compare **C** and **D**)~~ was closer to the upper boundary (**Fig. 4C**). ~~which~~ This could be due

to redundancy with another pathway that regulates invasive growth in this environment (i.e. **RAS**). Although an environment that triggers maximal invasive growth and **MAPK** pathway activity remains to be identified, these results suggest that the **MAPK** pathway typically operates at less than half its maximum levels of activity, and promotes invasive growth to less than half its maximal levels, even in environments where the pathway is activated to stimulate invasive growth. Thus, the **MAPK** pathway functions at modest levels to induce invasive growth…"

8) Figure 5H: Consider changing to have the arrows all point in the same direction like Figure 3C. For gene expression, I instinctively associated up arrows with up-regulation and down arrows with down-regulation.

We have made this change.

Response to Reviewer #2

Reviewer #2: This is an interesting paper that uses gene-by-environment analysis of invasive growth in yeast to identify conditional gene-gene and gene-phenotype relationships within the signaling network that controls the phenotype. This work has the potential to be of interest to a wide group of researchers interested in signaling network dynamics and the plasticity of networks under different environmental conditions.

Thanks very much to the reviewer for their helpful suggestions. We have taken these suggestions to heart by performing additional experiments and by performing extensive statistical tests. Thanks very much for these suggestions. We believe that the manuscript is clearer, more accurate, and very much improved.

I have two major concerns with the paper, one suggestion for increasing the impact of the work and a number of minor issues.

First major concern: I am not convinced that the invasive growth measurements fully control for confounding effects of growth. Invasive growth is measured as scar-intensity/colony area. Unless this ratio stays the same throughout colony growth then you could imagine the following scenario: Wild-Type has an invasion value of 2 at day-2 (small colony), value of 3 at day-5 (medium colony) and value of 4 at day 7 (large colony). Deleting gene X has an effect only on growth, so that colonies follow the same trajectory as WT but with a delay, so that at day 5 they have an invasion value of 2 (small colony) and at day 7 an invasion value of 3 (medium colony). Using the authors' approach, this would suggest that deletion of gene X causes lower than WT invasion at days 5 and 7 (ratios of 2/3 and ¾), when all that was really happening was reduced growth, with no effect on invasion behavior (relative to colony size). This line of thought suggests that normalization between genotypes or conditions should be done between colonies of the same size. Can the authors comment on this concern?

We understand and agree that changes in colony size/growth rate might complicate assessment of relative invasive growth among mutants, especially over time. We also agree that some mutants show growth defects (e.g. *snf1*), and some colonies grow at different rates on different conditions. For most experiments, comparisons are made under conditions where mutants all show the same growth rates. In the revised manuscript, we focus mainly on several key pathways (**MAPK, RAS, RIM101**, and **OPI1**). Mutants lacking functional pathways did not show differences in colony size over time (*Fig. S7*), which indicates that the growth rates of these mutants are similar.

We also carefully measured the growth rates of the mutants in liquid culture, under the main conditions tested. The mutants showed similar growth rates to wild-type cells, and we have included this data as a supplemental figure in the revised manuscript (see below):

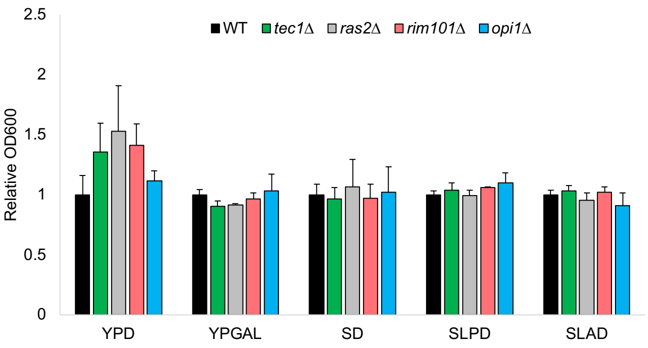

Figure S4. Growth of wild-type cells and mutants lacking the major regulatory pathways that control invasive growth. Wild-type cells and the indicated mutants were grown in the indicated media in liquid culture for 16h at 30˚ by shaking. Cells were washed once in water, and growth was measured by OD at 600nm. For each condition, wild-type values were set to a value of 1. The average of three replicates is reported. Error is reported as standard deviation.

We refer to the figure on P12: "…To further explore variations in invasive growth regulation, strains lacking the main regulators (**RAS, MAPK, RIM101,** and **OPI1**), which showed no growth defects compared to wild-type cells (*Fig. S4*), were re-examined across environments…"

Second major concern: I have a major concern with the statistical analyses. The research presented is exploratory and involves a large number of statistical tests (all t-tests, I believe), both explicitly presented in the figures and implicitly in the fact that the examples that are illustrated are derived from a much larger number of possible comparisons, with the choice of what to present likely driven by what appeared most significant. I can find no mention in the paper of correcting p-values for multiple hypotheses. If I am correct that no correction for multiple hypotheses was undertaken, then I am skeptical that many of the fairly modest effects discussed are actually statistically significant.

To address this concern, we have performed an extensive statistical analysis that corrects p-values for multiple comparisons for the plate-washing assay survey. We performed one-way ANOVA analysis to compare mutants to wild type (by Dunnett's multiple comparisons test) and to compare the mutants to each other or across environments (both by Tukey's multiple comparisons test). The results of these tests have been included as supplemental files (*Files S1-S3*).

After including the statistical data, we updated the M&M section: "…Significance was determined using the program GraphPad Prism (commercially available: https://www.graphpad.com/) by one-way ANOVA analysis unless otherwise indicated. Dunnett's multiple comparisons test was used for comparisons of mutants to wild type. Tukey's multiple comparisons test was used to compare mutants to mutants or a mutant across environments. Confidence interval was set to 95%. Reported p-values were adjusted for multiple comparisons. Statistical analyses can be found in *Files S1-S3*. ~~Student's t-test~~."

We also updated the figure legend for **Fig. 1D**: "…Raw data is shown in *Fig. S1*; Bar graphs with standard deviation in *Fig. S2*; and statistical tests that show significance of pairwise comparisons in *Files S1-S3*…"

In the revised manuscript, we refer to the statistical analysis where the data is initially discussed. Most of the comparisons did not change as a result of the statistical analysis, and we now include p-values when referring to comparisons. A few comparisons we no longer statistically significant, and these have been removed from the revised manuscript. We make sure that only statistically significant examples are discussed and have included accompanying p-values in the text.

Changes to the manuscript were as follows:

- P9: "…**Fig. 1**, **B** and **C**, full PWA in *Fig. S1, A* and *B*, statistical analysis in *File S1*…"

- P9-P10: "…invasive growth of mutants was first compared to wild-type (**Fig. 1D**, red, less invasive than WT; blue, more invasive, white, no difference, raw data *Fig. S1,* quantitation with standard deviation in *Fig. S2*, statistical analysis in *File S2*). Most mutants showed differences in invasive growth from wild type (**Fig. 1D**, e.g. YPD, tenth row, *ras2*Δ mutant showed decreased invasion relative to wild type, p-value <0.0001). Mutants also showed differences across environments (**Fig. 1D**, compare colors across rows, statistical analysis in *File S1*). For example, the *ras2*Δ mutant showed variable shades of red across environments (**Fig. 1D**, e.g. compare YPGAL to WL, p-value <0.0001, YPGAL to SLAD, p-value 0.04, and WL to SLAD, p-value 0.02). The mutants were also different from each other (**Fig. 1D**, compare colors down columns, statistical analysis in *File S3*). The *ras2*Δ mutant for example was not as critical as the *opi1*Δ mutant for invasive growth on ~~YPD~~ YPGAL medium (**Fig. 1D**, *ras2*Δ is a lighter shade of red, p-value 0.0006)…"

- P12-P13: "…However, in one environment (YP), **OPI1** had a minor role (*File S1*, p-value < 0.0001). The idea that a pathway can play a major role in some environments but not others was also true for **RIM101** (**Fig. 2A-C**, compare YP to WL, p-value 0.0001), **RAS** (**Fig. 2A-C**, compare WL to YPD, p-value 0.0005), and **MAPK** (**Fig. 2A-C**, compare YPGAL to SLPD, p-value < 0.0001)…"

- P14: "…Wild-type colonies had a ruffled pattern on some environments (**Fig. 1A,** YPGAL ~~YPD~~) but not others (**Fig. 1A,** SOE). The different patterns did not correspond to differences in invasive growth (**Fig. 1B**, compare YPGAL ~~YPD~~ to SOE, *File S1*, p-value 0.0015)…."

- P22: "…(**Fig. 5A**, full data set in *Fig. S1-S2*, statistical analysis in *Files S1-S3*)…"

- P24: "…Unexpectedly, some pathways played a larger role than Flo11p in several environments. The *tec1*Δ, ~~*rim101*Δ,~~ and *opi1*Δ mutants showed less invasion (e.g. YPGAL, *Fig. S1-S2*, *File S3*) and the *tec1*Δ, *rim101*Δ, and *opi1*Δ mutants were less adherent (**Fig. 5, E** and **F**) than the *flo11*Δ mutant…"

For the heat map (**Fig. 1D**, see below, top), we have changed the range of the color white from a value of 1 to values of 0.8-1.2 and increased the lower threshold to < 0.1. As a result, subtle effects on invasive growth have been removed. We also removed the WT row for simplicity. The heat map in (**Fig. 5A**, see below, bottom) was adjusted in the same way to match.

Original New

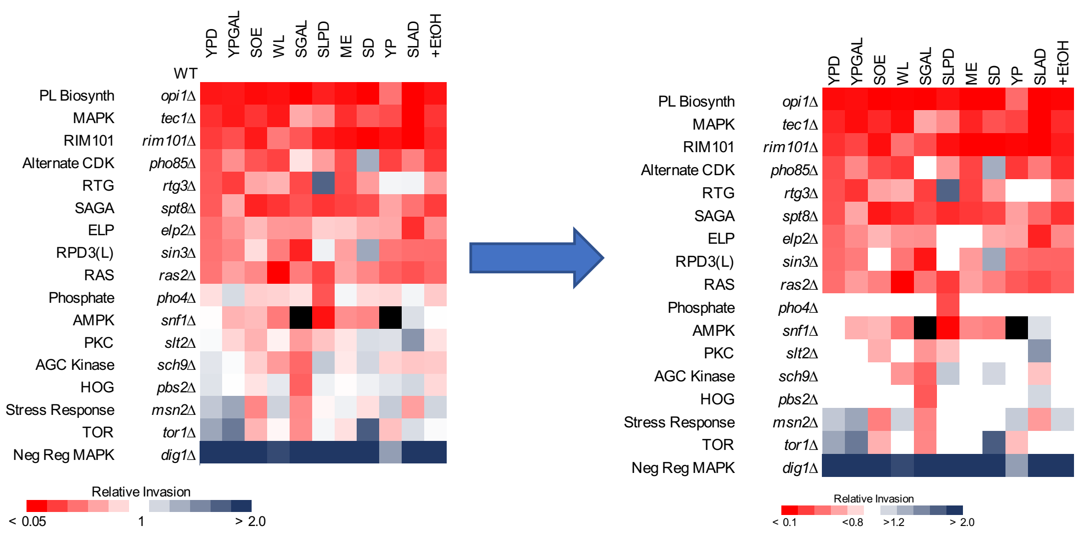

Original New

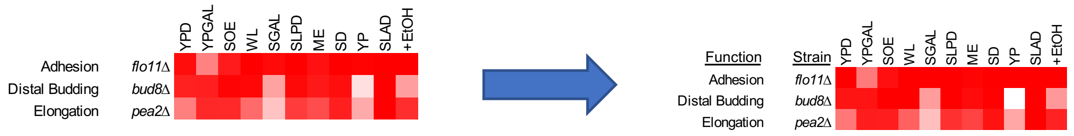

Increasing the impact: The paper does a thorough job of illustrating that gene-gene and gene-phenotype relationships can change between environments. There is also some illustration of how changes in gene expression of a small number of effector genes accompany these changes in environment and genotype. To make this paper of more general interest, the authors need more molecular data explaining HOW an environmental change changes gene-gene and gene-phenotype relationships, perhaps exploring expression levels and phosphorylation states of more genes in the signaling network, rather than just downstream effectors.

We agree and have performed preliminary qPCR and phosphoblot analysis to begin to define how environmental changes influence gene-gene and gene-phenotype relationships. Although we see gene-phenotype interactions by these tests, the mechanisms by which this regulation occurs is not yet clear. This question represents a new and interesting area of exploration for our lab and for the field for years to come, and we hope to address this issue thoroughly in a future study.

Minor Points:

The authors need to be more precise about the meaning of terms used, particularly as relating to "regulating" the signaling network. It is often unclear whether this means changing the network graph, or producing different outputs from a stable network because of different environmental inputs.

We agree and have made the following changes for clarity:

- ABSTRACT: "…How signaling networks ~~are regulated to~~ produce different phenotypes in different settings is not fully understood ~~clear~~…"

- AUTHOR SUMMARY: "…How networks ~~are regulated to~~ produce different…"

- AUTHOR SUMMARY: "…These insights into ~~the regulation of~~ network plasticity…"

- P26-P27: "…it revealed new aspects of ~~network~~ regulation occurring between pathways in the network, such as ~~conditional role reversals~~, conditional pathway redundancy and cross-pathway regulation; ~~and~~ it revealed the conditional regulation of gene expression for downstream targets of the signaling network; and it identified a new phenotype (round-cell invasion)…"

- P28: "…Collectively, ~~the plasticity in regulation~~ these results indicate ~~tells us~~ that…"

P5. "Phenotypic changes based on the environment are referred to as Gene (or Genotype) by Environment Interactions (GEIs)". This definition of GEI is incorrect (it does not include the effect of different genes/genotypes).

We have made the following changes on P5: "…Phenotype is generated from genotype, the environment, and interactions between the two [Gene (or Genotype) by Environment Interactions (GEIs)]. ~~Phenotypic changes based on the environment are referred to as Gene (or Genotype) by Environment Interactions (GEIs), and~~ GEIs are found ~~all~~ across all kingdoms of life…"

P9. "The fact that invasive growth regulatory pathways show different roles depending on environment is surprising, because the prevailing view is that the major pathways play an equal role, in that their loss causes a complete reduction in invasive growth" – I think this is a straw man and that this view is not generally held. In the absence of a reference clearly arguing that the role of the different pathways is equal, the authors should remove this claim.

We have removed the claim and made the following change on P10: "…~~The fact that invasive growth regulatory pathways show different roles depending on environment is surprising, because the prevailing view is that the major pathways play an equal role, in that their loss causes a complete reduction in invasive growth. The ability to see differences in~~ Because the role each pathway played in regulating invasive growth changed across ~~conditions~~ environments, ~~allowed us to compare pathways to each other~~ we asked whether some pathways play a major role in more environments than others, making them a stronger overall regulator of the biological process. To define the ~~relative roles~~ signaling pathways ~~play~~ with the strongest overall role in regulating invasive growth ~~a biological process~~, we ranked…"

We also made a change to the last paragraph of the INTRODUCTION: "…Specifically, we found pathways made different contributions to the regulation of invasive growth in different environments. We were able to ~~order~~ rank pathways by their overall contribution, ~~that were previously thought to all play the same roles, and we identified~~ which led to the identification of an underappreciated pathway as one of the main regulators of the response…"

P9. The ranking exercise the authors undertake is interesting, but they need to be clearer about its limitations. The set of conditions that were tested is only a very small subset of all possible environmental conditions, therefore the ranking is specific only for the set of conditions used and cannot be generalized to an absolute ranking of the pathways. In addition, describing Opi1p as the "main regulator of invasive growth" implies 'necessary and sufficient' (at least to me) perhaps something more cautious, like "strongest single regulator" would be better.

The following statement was removed from the figure legend of Figure 1E: "~~Ranking is specific to the strain background and environments tested. A different order may occur in different strain backgrounds and environments."~~

and was moved into the RESULTS section on P11: "Because invasion phenotypes can be influenced by genetic background [127-129], our ranking may be specific to the strain background (Σ1278b) and environments tested here."

Additionally, on P11 we made the following change: "…Opi1p as the ~~main overall~~ strongest single regulator of invasive growth..."

P9. "Invasive growth was also examined to identify the roles pathways and protein complexes play in regulating changes to invasive growth that occur when transitioning from one environment to another". The authors should rewrite this as no transitions actually occurred, instead invasion was simply compared between conditions for different mutants. Similarly, on P10 "when transitioning between certain environments".

As we discuss in the next point, we have removed the reaction norm data from the paper.

P10. I am not sure about the utility of Figure 1E. I have concerns about normalizing to YPD for the reaction norms (Figure 1E) or normalizing to any other condition (Figure S4). In terms of this study there is nothing special about YPD (or any other condition) that makes it an appropriate "base condition", instead it is simply one of several environments tested. For deletion strains where the normalization condition is an outlier, this will distort their entire profile across the remaining environments. This issue affects the whole discussion on P10. Instead, it would be safer to compare rows directly from Figure 1C to make arguments about how the importance and direction of effect of different genes (relative to WT) changes in different conditions. This is because WT is an appropriate baseline to compare the other genotypes to.

We agree and have removed the reaction norm data from Figure 1E and *Fig. S4*. We removed the discussion from P10. We removed reaction norms from the M&M. This has had the effect of streamlining the paper and focusing on the main points. We have instead included a brief discussion on the overall concept to P13 by referring to Figure 2A where wild type can act as a baseline to compare genotypes as the reviewer suggested. The changes are as follows:

"…We noticed when comparing two environments where wild type showed a change in invasion, some mutants showed the same change while other mutants showed no change. For example, wild-type cells showed increased invasion on WL medium compared to SLPD medium (**Fig. 2A**, **Fig.**

1C, statistics in *File S1,* p-value 0.0002) as did the *rim101*Δ mutant (**Fig. 2**, **A** and **B**, p-value 0.0005); however, the *opi1*Δ mutant showed a similar level of invasion (**Fig. 2**, **A** and **B**, p-value, n.s., 0.78). These data may suggest when a pathway is involved in sensing the difference between the environments (i.e. **OPI1** in this example) compared to a non-sensing role (i.e. **RIM101** in this example). Future studies aimed at testing these predictions by transitioning cells from one environment to another may uncover sensory mechanisms in the regulation of invasive growth."

P12 The second paragraph, beginning "The invasive growth network" makes contradictory statements about the set of genes regulating invasive growth vs complex colony morphology. I think the authors are arguing that the same gene network regulates both phenotypes, but that the relative importance and role of genes/pathways in the network differs between the above-surface and below-surface environments (presumably through different inputs into the signaling network). The authors need to clarify what they mean here.

We agree that as written the paragraph was unclear. We made the following changes to the paragraph on P14-P15:

"…~~The~~ Many pathways and effector genes in the invasive growth network also play a role in regulating ~~controls~~ complex-colony morphology [ruffling of cells above the surface [132]], which is an aspect of biofilm/mat formation [133]. Biofilm/mat formation also occurs in distinct patterns resulting from different microenvironments [134]. For example, wild-type colonies had a ruffled pattern on some environments (**Fig. 1A,** YPGAL ~~YPD~~) but not others (**Fig. 1A,** SOE). The different patterns did not correspond to differences in invasive growth (**Fig. 1B**, compare ~~YPD~~ YPGAL to SOE, *File S1*, p-value 0.0015). This result indicates that different regulatory mechanisms occur above and below the surface. Many pathways played a similar role in generating phenotypes above and below the surface (*Fig. S1*); however, at least one pathway ~~was found to~~ played different roles in these microenvironments (*Fig. S6*, **RTG**). This result supports the idea that biofilm/mat formation and invasive growth are regulated by both overlapping and non-overlapping ~~different~~ sets of pathways/genes [73]. Therefore, microenvironments reveal plasticity in the roles ~~regulatory~~ pathways play in regulating filamentous growth in different regions within a community of cells…"

P12. I'm not sure it is possible to determine roles in "initiating" and "maintaining" invasive growth using the results in Figure 2F. These results look consistent with a simple reduced rate of invasive growth in all of the mutants.

We made the following changes on P15-P16:

"~~We speculated that some pathways might be involved in the initiation of invasive growth, while others function at a later step to maintain the response.~~ We became interested in whether the pathways also differentially regulate invasive growth at different time points. To address this possibility, invasive growth was examined over time. In one environment (YPGAL), invasive growth increased over a three-week incubation (**Fig. 2F**, full PWA in *Fig. S7A*). All four pathways played a major role in regulating ~~were required for initiation of~~ invasive growth at an early time point tested (2d) but played different roles at different times (**Fig. 2**, **F** and **G**, YPGAL, 3d-21d). For example, **MAPK** was more critical at an early time point (3d) compared to a later time point

(21d) for invasive growth (**Fig. 2**, **F** and **G**). ~~In particular, **RAS** was less critical than the other pathways for maintaining invasive growth (3d-7d)~~. Interestingly, the pathways made ~~the~~ different contributions from one another at earlier time points (2d-7d) but the same overall contribution ~~to invasive growth by~~ at the last time point tested (21d) [**Fig. 2G**, YPGAL]. ~~although~~ However, the patterns were different (**Fig. 2F**, YPGAL). Therefore, ~~all of the main~~ major regulatory pathways ~~function during~~ can make varying contributions ~~the initiation of invasive growth but make unique contributions~~ during the progression of ~~the response~~ invasive growth.

GEI analysis was also used to explore the temporal development of invasive growth across environments. Like for YPGAL medium, on SD medium invasive growth increased over time (**Fig. 2F**, and *Fig. S7B*). However, pathways played different roles over time in the two conditions, based on the levels and patterns of invasive growth (**Fig. 2**, **F** and **G**, SD, and *Fig. S7C*). For example, ~~between~~ when comparing 7d to 21d, **RIM101** contributed similarly to invasive growth at both time points in one environment (YPGAL) but differently at both time points in another environment (SD) [**Fig. 2G**]. ~~**MAPK** was more critical for the progression of invasive growth on YPGAL, while **RIM101** was more critical on SD [compare green and red bars at 7d].~~ Moreover, ~~the~~ all four pathways did not equally contribute by 21d on SD like they did on YPGAL medium (**Fig. 2G**). Invasive growth after 21d also revealed GEIs for invasive growth patterns not seen at earlier time points between YPGAL and SD media ~~and the invasive patterns were different~~ (**Fig. 2F**, 21 d, e.g. **RIM101** was required for center-ring invasion on SD but not YPGAL). Thus, the temporal roles pathways play in the development of the response may change~~s~~ in different environments."

P13. Why does the fact that the sum of the mutant invasion scores is greater than 1 suggest redundancy?

The sentence has been removed (see next comment).

P13 Line304. I see very few instances where a pathway is required for invasion (OPI1 on WL medium, is one example) and many situations where pathways are partially dispensable.

We made the following changes to this part of the RESULTS on P16: "…~~We noticed that the relative invasion of the main pathway mutants summed to a higher value than wild type in some environments (**Fig. 2B**, YPGAL, sum of mutant relative invasion [1.28] is > 1). This observartion suggests that the pathways might exhibit redundancy. Moreover, In some settings multiple pathways were required for invasion (see **Fig. 2G**, 2d), yet on other environments, multiple pathways were partially dispensable [(**OPI1**, **MAPK**, and **RAS** on YP, **Fig. 2B**) and (**MAPK**, **RAS**, and **RIM101** on SGAL, *Fig. S2*)].~~ When regulating a phenotype, signaling pathways can make separate (i.e. additive) contributions from one another, or they can make overlapping (i.e. redundant) contributions. ~~Pathway redundancy is an underexplored facet of network regulation.~~ To test if the major pathways played additive or redundant roles ~~To more directly examine pathway redundancy~~ during invasive growth…"

P15. "Intriguingly, the environment where RTG played a negative role, PHO85p played a positive role and vice versa". Deletion of either reduces invasion in YPD, so this is only true for SD vs SLPD.

We changed the sentence for accuracy on P18:

"Intriguingly, when comparing SLPD to SD media, the environment where **RTG** played a negative role, **Pho85p** played a positive role and vice versa (**Fig. 3F**)."

P16. It would be more informative to see a scatterplot of MAPK activity against (WT) invasive growth in the different environments tested rather than trying to compare Figures 4C and 4D.

We have included a scatterplot in the revised manuscript (*Fig.* S7D).

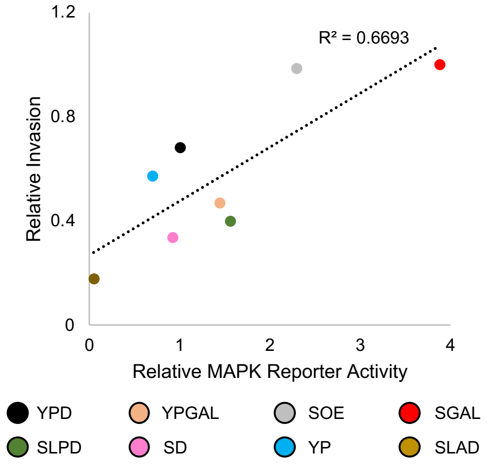

We also refer to the scatterplot data on P18:

"**MAPK** pathway activity correlated with invasive growth (*Fig. S7D*)."

We have also edited the manuscript on P19 to remove the comparison of 4C and 4D and improve clarity:

"One exception was on YP media, where invasive growth, but not ~~was similar to~~ **MAPK** pathway activity, ~~for most environments (**Fig. 4D,** compare **C** and **D**)~~ was closer to the upper boundary (**Fig. 4C**)."

P16 The use of the Tec1 and Dig1 deletions to define upper and lower invasive growth boundaries in Fig 4D is not explained.

We made the following changes to the manuscript on P19 to clarify:

"~~Similarly,~~ Like **MAPK** pathway activity, ~~to~~ **MAPK**-dependent invasive growth in wild-type cells ~~invasion~~ was closer to the lower boundary (represented by the hypoinvasive *tec1*Δ mutant) compared to the upper boundary (represented by the hyperinvasive *dig1*Δ mutant [*Fig. S1, Fig. S2B*]) in most environments tested…"

Based on another reviewer's comments, we have also made adjustments to the figure to improve clarity:

1. We have color coded the environments in 4A to match 4C and 4D.
2. We removed the additional invasive growth conditions in panel 4D so all conditions are the same for all panels in Figure 4.
3. We combined panels C and D into one figure and moved the key down to the bottom of the panel.
4. We added labels for "Min MAPK" and "Max MAPK"

P20 +21. I am not sure that expression levels of SFG1 are a good proxy for FLO11-independent adhesion mechanisms, particularly because SFG1 also regulates FLO11.

In previous work (Vandermeulen and Cullen, 2020), we have shown that Sfg1p mainly regulates invasive growth separately from Flo11p. Nevertheless, this is a good point that we now address in the revised manuscript on P24:

"Sfg1p, along with other transcription factors [161-163], regulates daughter-cell separation [164], has a minor role in regulating the expression of *FLO11* [103], and mainly contributes to invasive growth by an adhesion mechanism that is separate from Flo11p [103]."

P23. Describing OPI1 as a "top regulator" implies it is upstream of all the pathways, not that it has the single strongest effect on the phenotype.

We agree and changed "a top regulator" to "a major regulator" in the two places where it was discussed: P8 heading of first RESULTS section and P26 of the DISCUSSION.

P26 What does "mutant phenotype analysis" refer to that is different from GEI?
P26 The sentence beginning "GEI analysis…" is very opaque and the authors should clarify their meaning

The sentence has been rewritten as follows on P31:

"GEI analysis~~, when combined with mutant phenotype analysis,~~ can reveal new fundamental insights (and new phenotypes) by capturing ~~the pleiotropic nature~~ the different environmental roles of regulatory pathways and preventing the characterization of only environment-specific functions…"

Response to Reviewer #3

Reviewer #3: -Overall very interesting dataset that will be of interest to this community. I believe the data merit strong consideration for publication, if care is taken to correct issues in the presentation of that data. In particular, I felt that the paper was careless in its claims of novelty for some of its findings. My concerns are further spelled out below.

Thanks very much to the reviewer for their helpful suggestions. We have thought carefully about these points raised and made many changes to the manuscript, which have improved the paper and made the conclusions clearer.

-Strong statements throughout about prior knowledge in the field. I don't agree that all the reported findings are new, for instance some of what they claim is new is spelled out in the review Bruckner 2012, which is cited. A few example lines are below. More broadly, statements like "How networks are regulated to produce different phenotypes in different environments is not well understood" in the abstract could use justification. Much work in molecular biology has focused on how pathways respond to environmental cues.
"These results indicate that invasive growth is not binary (ON/OFF) but occurs in a phenotypic spectrum based on the environment." [182]
"the prevailing view is that the major pathways play an equal role, in that their loss causes a complete reduction in invasive growth" [201]
"In any case, this remarkable finding demonstrates that the same phenotype can arise through the action of pathways that have opposing roles in different settings." [Line 342]

We agree and have made the following changes:

- ABSTRACT: "…How signaling networks ~~are regulated to~~ produce different phenotypes in different settings is not fully understood ~~clear~~…"
- INTRODUCTION: "…~~An emerging view of~~ Signaling pathways ~~is that they~~ do not operate independently, and instead function ~~together along~~ with other pathways…"
- INTRODUCTION: "…which show ~~can provide a glimpse of~~ the comprehensive nature…"
- INTRODUCTION: "…We also identified a ~~new~~ round-cell invasion phenotype…"
- INTRODUCTION, last paragraph: "…~~Remarkably,~~ Invasive growth has ~~only~~ mainly been studied under ~~a few~~ select laboratory conditions, ~~even though it is triggered by diverse stimuli~~. However, *S. cerevisiae* is a free-living…"
- INTRODUCTION: "…we gained ~~entirely~~ new insights into the invasive growth regulatory network…"
- P9: "These results ~~indicate~~ support the existing idea that invasive growth is not binary (ON/OFF) but occurs in a phenotypic spectrum based on the environment."

- P10: "…~~The fact that invasive growth regulatory pathways show different roles depending on environment is surprising, because the prevailing view is that the major pathways play an equal role, in that their loss causes a complete reduction in invasive growth. The ability to see differences in~~ Because the role each pathway played in regulating invasive growth changed across ~~conditions~~ environments, ~~allowed us to compare pathways to each other~~ we asked whether some pathways play a major role in more environments than others, making them a stronger overall regulator of the biological process. To define the ~~relative roles~~ signaling pathways ~~play~~ with the strongest overall role in regulating invasive growth ~~a biological process~~, we ranked…"

- P16: "In any case, this ~~remarkable~~ finding ~~demonstrates~~ supports the idea that the same phenotype can arise through the action of pathways that have opposing roles in different settings."

- P18: "…~~Pathway redundancy is an underexplored facet of network regulation~~…"

- P22: "…The three effector phenotypes have some independence from each other in regulating invasive growth [159, 160]; however, whether adhesion, distal budding, and cell elongation ~~play an equal role during invasive growth, and whether their~~ change their contribution to invasion ~~changes~~ across environments is unknown…"

- P22: "…We found each aspect of invasive growth contributed differently from one another, in agreement with previous studies [159, 160], and their contribution changed based on the environment…"

- P26: "…Our approach uncovered ~~entirely~~ new insights into the network…"

- P26: "…It allowed us to rank the pathways in the network ~~which identified~~ and assign an underappreciated regulator as a new ~~top~~ major regulator …"

- P27: "…GEI analysis also allowed interrogation of the flexible and invariant components of the network and ~~nullified the hypothesis that all major pathways play an equal role. GEI analysis~~ showed that the major…"

- P30: "…An ~~important~~ exception to decentralized control…"

- P31: "…can reveal new ~~fundamental~~ insights…"

-Definition of GEI in line 111 seems off. Shouldn't that be about variant genotypes interacting with the environment, not just phenotypes changing due to environment?

We have made the following change on P5:

"Phenotype is generated from genotype, the environment, and interactions between the two [Gene (or Genotype) by Environment Interactions (GEIs)]. ~~Phenotypic changes based on the environment~~

~~are referred to as Gene (or Genotype) by Environment Interactions (GEIs), and~~ GEIs are found ~~all~~ across all kingdoms of life…"

-Further, I am not sure I came away clear on what GEI analysis is. There is not a clear definition of this as an approach. Line 156 ("Coupling GEI analysis to conventional mutant analysis") implies that the authors are treating GEI analysis as something other than exposing mutants to different environments, which is how I would define GEI analysis.

We clarified this sentence by making the following changes on P8: "~~This broad-based survey revealed an unexpected diversity in the regulation of invasive growth. Coupling GEI analysis to conventional~~ By analyzing mutants on different environments (i.e. GEI analysis) in a broad-based survey ~~analysis~~, which is ~~has proven to be~~ a ~~powerful~~ useful approach…"

-The use of "reaction norms" starting at line 220 could use expansion. Materials and methods were insufficient in describing what this is or what it can tell you that is distinct from a standard normalization procedure.

We have removed the reaction norm data in response to another reviewer's comments. As a result, the manuscript is more focused and easier to read.

-In line 253, "invasion scar" is used for the first time without a definition. Could go in the introduction when defining the phenotype.

We added the word 'invasive' to the introduction of the PWA on P9 for clarity.

"…where a community of cells washed off a surface leaves a visible invasive scar…"

-Work from the Ehrenreich lab (and others) has shown that mutations related to invasion phenotypes are strongly influenced by genetic background. It would be good to point this out, as some of the findings, like the dominance of the OPI pathway, may be background-specific. A few lines on this are in the figure 1 legend, but should be located somewhere in the main text instead and would benefit from more discussion.

The following statement was moved from the figure legend of Figure 1E: "~~Ranking is specific to the strain background and environments tested. A different order may occur in different strain backgrounds and environments.~~"And moved to the body on P11 with new references as follows: "Because invasion phenotypes can be influenced by genetic background [127-129], our ranking may be specific to the strain background (Σ1278b) and environments tested here."

The three references added are new and as follows:

- Matsui T, Linder R, Phan J, Seidl F, Ehrenreich IM. Regulatory Rewiring in a Cross Causes Extensive Genetic Heterogeneity. Genetics. 2015;201(2):769-77. Epub 2015/08/02. doi: 10.1534/genetics.115.180661. PubMed PMID: 26232408; PubMed Central PMCID: PMCPMC4596683.

- Song Q, Johnson C, Wilson TE, Kumar A. Pooled segregant sequencing reveals genetic determinants of yeast pseudohyphal growth. PLoS Genet. 2014;10(8):e1004570. Epub 2014/08/22. doi: 10.1371/journal.pgen.1004570. PubMed PMID: 25144783; PubMed Central PMCID: PMCPMC4140661.

- Chin BL, Ryan O, Lewitter F, Boone C, Fink GR. Genetic variation in Saccharomyces cerevisiae: circuit diversification in a signal transduction network. Genetics. 2012;192(4):1523-32. Epub 2012/10/12. doi: 10.1534/genetics.112.145573. PubMed PMID: 23051644; PubMed Central PMCID: PMCPMC3512157.

-The mix of transcription factors and upstream regulators may also impact the results regarding the ranking of pathways. When Ras2 is knocked out, its downstream regulators are still present, whereas with OPI, the actual TF is gone. Adding in some additional mutants in genes in different locations withing these signaling pathways would be great, if possible. At the least discussion of the possible impacts of these choices is essential in my view.

In previous studies, multiple pathway components have been examined in isogenic strains. Cells lacking Ras2p and the transcription factor Flo8p have the same phenotype in invasive growth. Cells lacking Ste20p, Ste11p, Ste7p, and Ste12p have the same phenotypes. Cells lacking Rtg1p, Rtg2p, and Rtg3p have the same phenotype, and cells lacking Rim8p and Rim101p have the same phenotype. We reference these results in the revised manuscript on P9-P10:

"…Genes were chosen that ablate pathway activity based on previous tests comparing mutants that lack key pathway components [86, 88, 124]…"

-Many interpretive statements would benefit from being moved to the discussion, such as line 372 "Perhaps other pathways operate at low overall levels as a safeguard to curb pathway activity. It is clear at least for the ERK pathway that overactivation can lead to cancer and other diseases."

- The sentences on P19-P20: "…~~The fact that **MAPK** pathway activity functions at modest levels to induce invasive growth is consistent with previous results from our laboratory. We previously showed that the **MAPK** pathway induces expression of genes encoding multiple negative regulators of invasive growth and a negative regulator of **MAPK** activity (*NFG1*), which has the effect of modulating the response [103]. Curbing invasive growth is advantageous in the balance between invasion and colony expansion [127]. Curbing **MAPK** pathway activity is advantageous in that pathway hyperactivation leads to defects in budding and cell morphology [87, 122, 128]. Perhaps other pathways operate at low overall levels as a safeguard to curb pathway activity. It is clear at least for the ERK pathway that overactivation can lead to cancer and other diseases [129]…~~"

  Were moved to the DISCUSSION on P29 as follows: "…The fact that **MAPK** pathway activity functions at modest levels to induce invasive growth is consistent with previous results from our laboratory. The **MAPK** pathway induces expression of genes encoding multiple negative regulators of invasive growth and a negative regulator of **MAPK** pathway activity (*NFG1*), which has the effect of modulating the response [123]. Curbing invasive growth is advantageous in the balance between invasion and colony expansion [100]. Curbing **MAPK** pathway activity may be advantageous in that pathway

hyperactivation leads to defects in budding and cell morphology [88, 145, 150]. Perhaps other pathways operate at low overall levels as a safeguard to curb pathway activity. It is clear at least for the mammalian RAS-MEK-ERK pathway that hyperactivation can lead to cancer and other diseases [151]…"

- The statement "…~~Conditional redundancy may apply to all types of proteins and suggests complete redundancy between proteins should not be assumed if only tested in limited~~ environments…" was moved to P28 in the DISCUSSION as follows: "…Several pathways showed conditional redundancy with other pathways. Conditional redundancy implys~~ing~~ pathway interactions within the network are conditional and may apply to all types of proteins in networks. Perhaps this may suggest redundancy in protein function is not conserved for robustness or genetic buffering, but instead is conserved because non-redundant functions exist in some environments. In line with the finding of conditional redundancy ~~this discovery~~, cross-pathway regulation was conditional…"

-Figure 6b on round cell invasion: I'm not sure I see it like the authors. The cross section looks like a mix of elongated and round cells. Could it be that only some cells elongate to invade, and some incidental round cells hang around after washing due to expression of flocculins?

We have included new data (*Fig. S16*) that shows squashes of invaded cells at 100X magnification. Under this condition, most wild-type cells are round, and all cells of the *tec1* mutant are round, despite the fact that they show significant invasion into the agar substrate.

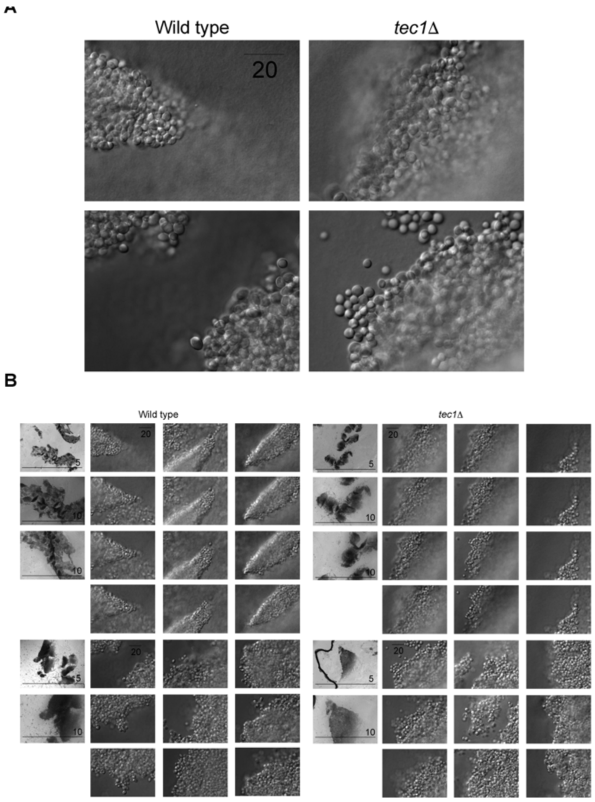

Figure S16. Invasive squashes to identify morphologies of cells undergoing invasive growth. **A)** Examples of invaded cells for wild-type cells and the *tec1*Δ mutant. Cells were visualized by microscopy at the 100X objective. Bar, 20 microns. **B)** Complete raw data of invasive squashes of wild-type cells and the *tec1*Δ mutant. Cells were visualized by microscopy at the 5X, 10X, and 100X objectives. Bars, 5, 10, and 20 microns as indicated. Several examples are shown from different squashes.

We refer to the new figure on P26: "…Cross sections of the invasive scar showed sub-surface invasion (**Fig. 6B**, cross sections, full imaging in *Fig. S15*) and agar squashes of embedded invasive cells revealed a round-cell morphology (*Fig. S16*)…"

The following text has also been included into the M&M at the end of the plate-washing assay section:

Invasive scar cross sections were performed by spotting equal concentrations of wild-type cells and the *tec1*Δ mutant onto SGAL medium. Plates were incubated for 7 d at 30˚C. Cells were washed off of plates in a stream of water. A razor blade and sterile forceps were used to excise the cells that had become embedded in the agar due to invasive growth. Chunks of agar containing invaded cells were placed on microscope slides to which a coverslip was added. For invasive squashes, gentle pressure was applied to squash the agar, revealing the trapped invaded cells. Cells were visualized by microscopy using the 5X, 10X, and the 100X objectives.

-Two papers that may be of interest came to mind while reading this. I believe they may have useful information for further interpreting your results:

1. Reynolds, T. B., Jansen, A., Peng, X. & Fink, G. R. Mat Formation in Saccharomyces cerevisiae Requires Nutrient and pH Gradients. Eukaryot Cell 7, 122–130 (2008).1.
2. Váchová, L. & Palková, Z. How structured yeast multicellular communities live, age and die? FEMS Yeast Res 18, (2018).

Thank you for bringing these great references to our attention. We have added the references to the manuscript.

The first reference was added:

- on P14 in the following sentences: "…Biofilm/mat formation also occurs in distinct patterns resulting from different microenvironments [110]."

- on P23: "…and Flo11p [108, 110, 159]…"

- on P30: "…This may reflect differences in pH or glucose concentrations in the different environments, which exist in gradients from the center of a community of cells to the outside rim [110]."

The second reference was added to P13 in the following sentence: "…Cells growing within microbial communities experience different microenvironments. Microenvironments can trigger specific responses in subsets of cells throughout the community [132].…"

-Again, this is a very exciting dataset and I hope that these comments are constructive.

Thanks again for your supportive comments.

We have also made the following additional changes to the manuscript:

Changes were made to the ABSTRACT for clarity.

Changes were made to the AUTHOR SUMMARY for clarity.

We added quantification of the *dig1* mutant on +KCl to Figure. S2B

We have adjusted the ACKNOWLEDGEMENTS and added Christian Landry, who has provided constructive input.

The following changes to the INTRODUCTION were made for clarity:

- "~~GEI analysis~~ Moreover, analyzing GEIs of signaling pathways may be a ~~useful~~ straight forward approach to employ in other systems to learn more about the regulation of complex phenotypes."

- "…which reveal dozens to hundreds of interacting proteins [55, 56], mathematical modeling to understand network fidelity and integration [57, 58], and increasingly by systems-wide approaches ~~analyses~~ [59-61], which show ~~can provide a glimpse of~~ the comprehensive nature of these web-like networks ~~complex systems~~…"

- "…connected to each other at multiple levels to ~~in ways that~~ coordinate…"

- The following struck through sentences were moved from the RESULTS on P21-P22 to the INTRODUCTION: "…~~Numerous genes are involved in the effector phenotypes, including *FLO11*, which encodes the major cell adhesion protein [92-99]; *BUD8*, which encodes a bud-site-selection protein that marks the distal pole [100-103]; and *PEA2*, which encodes a component of the polarisome, which is required for actin polymerization and cell elongation [104-109].~~…"

The following changes to the RESULTS were made:

- The following sentence was changed on P8 for clarity: "…including standard laboratory medium [YPD, yeast extract, peptone, dextrose], and media with varying amounts and/or sources of carbon, nitrogen, and phosphate, ~~and~~ including media thought to mimic a natural environment…"

- The following sentence was changed on P8 for clarity: "…environment ~~mimicking~~ representing a natural setting (SOE)…"

- The following sentence was changed on P10 for clarity: "…Opi1p is a positive regulator of invasive growth and biofilm/mat formation [125] and functions as a transcriptional repressor of phospholipid biosynthesis [126-128], but has not been appreciated as a major regulator of the response…"

- The following sentence was added to P13-P14 for clarity: "Wild-type cells showed different invasion patterns in different environments (**Fig. 2**, **D** and **E**, black line, top and bottom panels). We examined how the major pathways might affect the regulation of invasive growth in different microenvironments. Cells lacking the main regulatory pathways also showed different patterns."

- The following sentence on P19: ~~Although the environment that triggers maximal invasive growth may have yet to be identified, it is clear that the **MAPK** pathway is not maximally activated in environments in which cells undergo robust invasive growth.~~ was moved to the end of the section and changed to the following for clarity: "…Although an environment that triggers maximal invasive growth and **MAPK** pathway activity may remain to be identified, these results suggest that the **MAPK** pathway typically operates at less than half its maximum levels of activity, and promotes invasive growth to less than half its maximal levels, even in environments where the pathway is activated to stimulate invasive growth. Thus, the **MAPK** pathway functions at modest levels to induce invasive growth…."

- The following sentences on P22 were changed for clarity and accuracy: "…*FLO11*-dependent adhesion ~~was the~~ played a major role in ~~main contributor to~~ invasive growth in ~~some~~ most environments (**Fig. 5**, **A-C**), but not all ~~(YPD, YP, SLAD, +EtOH) but not others~~ (YPGAL ~~and SOE~~) ~~[**Fig. 5**, **B-D**]~~. Similarly, distal budding and cell elongation ~~was the~~ played major roles in ~~main contributor in one~~ several environments (**Fig. 5**, **A-C**, e.g. YPGAL and SOE) and minor roles in others (e.g. YP and SGAL). Each aspect behaved differently than the other aspects in at least one environment (**Fig. 5D**, *PEA2* on YPD, *FLO11* on YPGAL, *BUD8* on +EtOH). ~~and cell elongation contributed less than distal budding and adhesion in some environments (YPD) and more than distal budding (+EtOH) or adhesion (YPGAL) in others (**Fig. 5**, **B-D**). In one environment, distal budding and cell elongation were dispensable (**Fig. 5, B-D,** YP)~~…"… "…Therefore, one explanation for the diversity in invasive growth induced by the signaling network ~~can be explained by~~ comes from differences in the roles of effector processes…"

- The following sentence on P24 was changed for clarity: "…**RAS** regulates *SFG1* expression [83, 86], ~~and at this promoter~~ perhaps through the transcription factors Flo8p, Sok2p, and Phd1p, which bind by CHIP [84]…"

- The following sentence on P25 was changed for clarity: "…Such regulation may account for the phenotypic plasticity of invasive growth ~~diversity of phenotypes seen across environments~~…"

- The following sentences on P25 for accuracy: "…by ~~sustaining~~ regulating expression of *CLN1…"* … "…The fact that strains that cannot produce elongated cells ~~the *tec1*Δ and~~

~~*pea2*Δ~~ ~~mutants~~ exhibit invasive growth on most environments (*Fig. S1-S2*, *tec1*Δ and *pea2*Δ)…"

The following changes were made in the DISCUSSION:

- P26-P27 for clarity: "…Our approach uncovered ~~entirely~~ new insights into the network that regulates invasive growth. It allowed us to rank the pathways in the network ~~which identified~~ and assign an underappreciated regulator as a new ~~top~~ major regulator; it uncovered conditional role reversals for some regulators; it revealed new aspects of ~~network~~ regulation occurring between pathways in the network, such as ~~conditional role reversals~~, conditional pathway redundancy and cross-pathway regulation; ~~and~~ it revealed the conditional regulation of gene expression for downstream targets of the signaling network; and it identified a new phenotype (round-cell invasion). Given that many of the signaling pathways that regulate invasive growth are evolutionarily conserved, these finding may be broadly applicable to signaling networks in general…"

- P27-P28 for accuracy and ease of reading: "…different environments, microenvironments, and over time ~~revealing unexpected diversity in network plasticity~~. Most pathways were at least partially dispensable under ~~at least~~ one or more environments revealing unexpected network plasticity. ~~and~~ Several pathways showed conditional redundancy with other pathways …"

- The following sentence was changed on P28 to expand the conclusion: "…How this type of regulation occurs is not clear ~~and future work will be required to understand this type of regulatory mechanism~~. It will be interesting to see if conditional role reversals occur for one or all phenotypes a pathway regulates."

- The following sentence was changed on P29 for clarity: "…~~It has long been understood that~~ Many pathways independently converge on the *FLO11* promoter. We show here ~~we show~~ that *SFG1* is regulated by the major filamentation regulatory pathways as well. ~~Remarkably,~~ at least two pathways…"

- The following sentence was changed on P29 for clarity: "…**MAPK** pathway is the central regulator of polarity and cell shape ~~differentiation aspects of the response~~…"

The following additional references were added to the paper:

- On P5: Sardi M, Krause M, Heilberger J, Gasch AP. Genotype-by-Environment-by-Environment Interactions in the Saccharomyces cerevisiae Transcriptomic Response to Alcohols and Anaerobiosis. G3 (Bethesda). 2018;8(12):3881-90. Epub 2018/10/12. doi: 10.1534/g3.118.200677. PubMed PMID: 30301737; PubMed Central PMCID: PMCPMC6288825.

  To the following sentence on P5: "…fungi [10-20]…"

- On P27: Sreenivas A, Carman GM. Phosphorylation of the yeast phospholipid synthesis regulatory protein Opi1p by protein kinase A. J Biol Chem. 2003;278(23):20673-80. Epub 2003/04/02. doi: 10.1074/jbc.M300132200. PubMed PMID: 12668681

  To the following sentence on P27: "…Opi1p is a transcriptional repressor of phospholipid biosynthesis and inositol production [106, 107, 174, 175]."

- On P30: Clark AG, Vignjevic DM. Modes of cancer cell invasion and the role of the microenvironment. Curr Opin Cell Biol. 2015;36:13-22. Epub 2015/07/18. doi: 10.1016/j.ceb.2015.06.004. PubMed PMID: 26183445

  To the following sentence on P30: "…Microenvironments also play a major role in diseases like cancer [56, 206-208], including cancer cell invasion into new environments [209]…"
